# An Exposure to the Oxidized DNA Enhances Both Instability of Genome and Survival in Cancer Cells

**DOI:** 10.1371/journal.pone.0077469

**Published:** 2013-10-17

**Authors:** Svetlana V. Kostyuk, Marina S. Konkova, Elizaveta S. Ershova, Anna J. Alekseeva, Tatiana D. Smirnova, Sergey V. Stukalov, Ekaterina A. Kozhina, Nadezda V. Shilova, Tatiana V. Zolotukhina, Zhanna G. Markova, Vera L. Izhevskaya, Ancha Baranova, Natalia N. Veiko

**Affiliations:** 1 Research Centre for Medical Genetics, Russian Academy of Medical Sciences, Moscow, Russia; 2 V.A.Negovsky’ Research Institute for General Reanimatology, Russian Academy of Medical Sciences, Moscow, Russia; 3 Center for the Study of Chronic Metabolic Diseases, School of System Biology, George Mason University, Fairfax, Virginia, United States of America; ENEA, Italy

## Abstract

**Background:**

Cell free DNA (cfDNA) circulates throughout the bloodstream of both healthy people and patients with various diseases and acts upon the cells. Response to cfDNA depends on concentrations and levels of the damage within cfDNA. Oxidized extracellular DNA acts as a stress signal and elicits an adaptive response.

**Principal Findings:**

Here we show that oxidized extracellular DNA stimulates the survival of MCF-7 tumor cells. Importantly, in cells exposed to oxidized DNA, the suppression of cell death is accompanied by an increase in the markers of genome instability. Short-term exposure to oxidized DNA results in both single- and double strand DNA breaks. Longer treatments evoke a compensatory response that leads to a decrease in the levels of chromatin fragmentations across cell populations. Exposure to oxidized DNA leads to a decrease in the activity of NRF2 and an increase in the activity of NF-kB and STAT3. A model that describes the role of oxidized DNA released from apoptotic cells in tumor biology is proposed.

**Conclusions/Significance:**

Survival of cells with an unstable genome may substantially augment progression of malignancy. Further studies of the effects of extracellular DNA on malignant and normal cells are warranted.

## Introduction

Cell free circulating DNA (cfDNA) fragments can be collected from plasma, serum or other bodily fluids of both healthy people and patients with various diseases. Most often, the effects of cfDNA are studied using *in vitro* models of extracellular DNA (ecDNA), isolated from cell-free supernatants of cultured cells [1], either intact or exposed to various types of oxidative stress.

Oxidative stress is known to induce cell death. Dying cells release fragments of oxidized DNA into the cfDNA pool. cfDNA circulates throughout the body and causes secondary, systemic effects in distant organs and tissues. cfDNA extracted from blood plasma of patients with high oxidative stress levels is known to influence the physiological activity of intact cells [1-6]. In mesenchymal stem cells (MSCs), both ecDNA collected from the media of primary tumor cells cultures and cfDNA extracted from plasma of cancer patients have influenced ROS production [5]. In fibroblasts, oxidized ecDNA evokes an adaptive response that manifests as an increase in the resistance of treated cells to irradiation and chronic stress agents [7]. In fact, ecDNA fragments serve as stress signals for both the adaptive response and for bystander effect that develop in response to low dose irradiation in many types of cultured cells [1,8–15]. 

Previous *in vitro* studies profiled the various effects of cfDNA/ecDNA in cultured primary cells, including human endotheliocytes [2,3], mesenchymal stem cells (MSCs) [5,6], lymphocytes [8-10,12] and fibroblasts [7] as well as rat cardiomyocytes [4] and neurons[16]. However, no studies so far have described the effects of ecDNA on tumor cells, despite the obvious relevance of this model to the therapy of human malignancies, particularly due to the abundance of published observations indicating an increase in cfDNA concentrations in the circulation of cancer patients [17-25]. Cancer cells differ from normal ones by its increased levels of ROS; the levels of oxidation in tumor DNA are also higher that in the normal tissue. Indeed, both irradiation and chemotherapy lead to the oxidative death of large numbers of tumor cells, theoretically, resulting in a massive release of oxidized cfDNA. 

In this study, we describe the effects of increases in ecDNA oxidation and ecDNA concentrations on various characteristics of oestrogen (ER) and progesterone receptor (PR) positive breast carcinoma cell MCF-7. Here we show that oxidized ecDNA induce in these cells an oxidative stress that, on the one hand, is accompanied by a failure to maintain the stability of the genome and, on the other hand, leads to the development of adaptive response that enhances cell survival. 

## Results

Concentrations of ecDNA in the media conditioned by intact MCF-7 cells were, on average, at 140 ± 20 ng/mL. Effects of gDNA and gDNA^OX^ were evaluated after adding various concentrations of respective DNA to the cultivation media. Intact gDNA was extracted from primary human embryonic fibroblasts (HEFs), while gDNA^OX^ samples were obtained as a result of the treatment of gDNA with H_2_O_2_ as we described before [15]. Levels of 8- oxodG in gDNA were at ^~^0.1 8-oxodG per one million of 2'- deoxynucleosides, while in gDNA^OX^ these levels were at~750 8-oxodG per one million of 2'- deoxynucleosides [5,7]. To ensure that gDNA matches gDNA^OX^ by mean length of its fragments and their size distribution (0.2 to 15 kb), gDNA was treated with various concentrations of DNAse I and the matching gDNA sample was selected after electrophoretic evaluation in agarose gels. Comparative effects of gDNA and gDNA^OX^ treatments were studied at final media concentrations of 50 ng/mL or 5 ng/mL, while exposure varied from 30 minutes to 48 hours.

### 1. Localization of gDNA and gDNA^OX^ in MCF-7 cells

**Figure 1:**
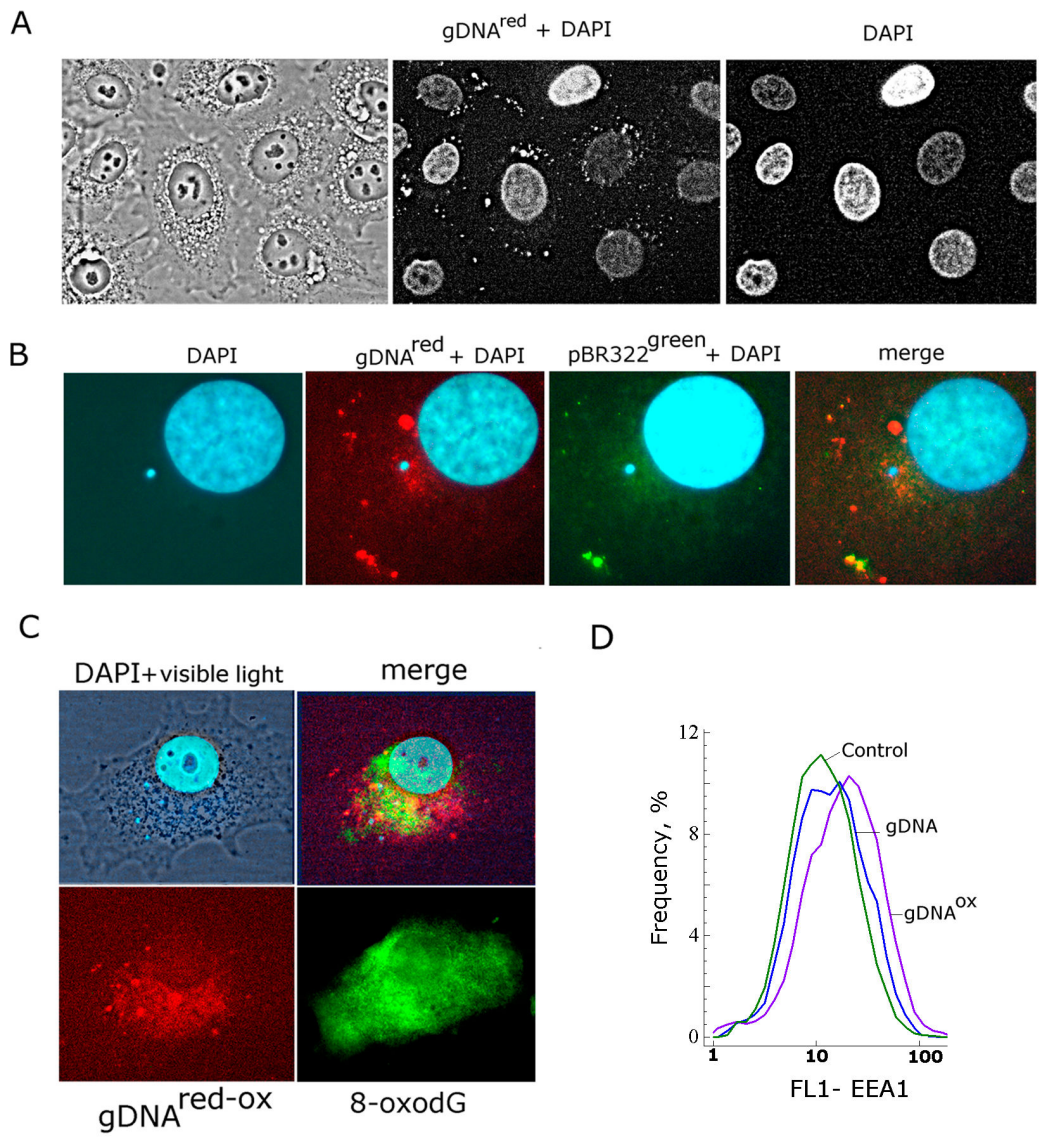
Staining of MCF7 cells with various types of labeled DNA. A - gDNA^red^, nuclei are stained with DAPI (x40); B – merged staining patterns of gDNA**^red^** and pBR322^green^ (x200); С – merged staining patterns of gDNA**^red-ox^** and FITC-conjugated antibodies to 8-oxodG (x200); D – FACS analysis of early endosomal marker EEA1; the distribution of the cells with varying EEA1 contents. Final concentrations of added DNA in the media were at 50 ng/mL; cells were incubated with DNA for 30 min before fixation in 3% formaldehyde. In case of staining with FITC-conjugated antibodies to 8-oxodG, fixed cells were pretreated with 0.1% Triton Х100 for permeation.

To find out the intracellular locations of gDNA and gDNA^OX^, a number of DNA probes were synthesized and differentially labeled. gDNA^red^ and pBR322^green^ probes were labeled using nick-translation with SpectrumRed and SpectrumGreen, respectively. In MCF-7 cells, gDNA^red^ and pBR322^green^ demonstrate similar granulated, clumped staining patterns in the periphery of the cytoplasm, visible in approximately 70% of cells (Figure 1А). More detailed analysis showed that intracellular distribution of labeled DNA fragments is sample specific (Figure 1В). In cells treated with both gDNA^red^ and pBR322^green^, some areas of the cytoplasm are stained with one, but not the other type of labeled DNA. Areas stained with more sequence-diverse gDNA^red^ are present in larger numbers and occupy a larger volume of the cell. In gDNA^red^ stained cells there was also a diffuse staining near the nuclear envelope that was visible at a higher magnification (x 200). Our observations indicate that **at** least some exogenous gDNA fragments are imported into the cell.

To determine the intracellular locations for gDNA^OX^, a composite probe was produced by slow renaturation of nick-translation labeled gDNA^red^ and gDNA^OX^ (gDNA^red-OX^). Similar to gDNA^red^, this composite labeled probe was also located at the periphery of the cytoplasm (Figure 1С), however, in case of the composite probe gDNA^red-OX^, a substantial portion of the labeled fragments were found inside of the cytoplasm near the nucleus. To confirm that this diffuse staining corresponded to oxidized DNA, we stained the cells with FITC-conjugated antibodies to 8-oxodG (Figure 1C). Our data indicate that gDNA^OX^ is imported into the cell at a substantially larger degree than gDNA. After entering the cell, gDNA^OX^ locates in the cytoplasm, forming foci around the nucleus.

Endocytosis is one of the common ways of delivery of exogenous compounds into the cell. The formation of novel endosomes is accompanied by an increase in expression of early endosome antigen 1 protein (EEA1), known as an early endosomal biomarker [26]. Using FACS, we demonstrated that exposure to DNA^OX^ leads **to an increase** of the proportion of cells that express high levels of EEA1 (Figure 1D). These observations are in concert with visual patterns of intracellular staining for gDNA^OX^.

**Figure 2:**
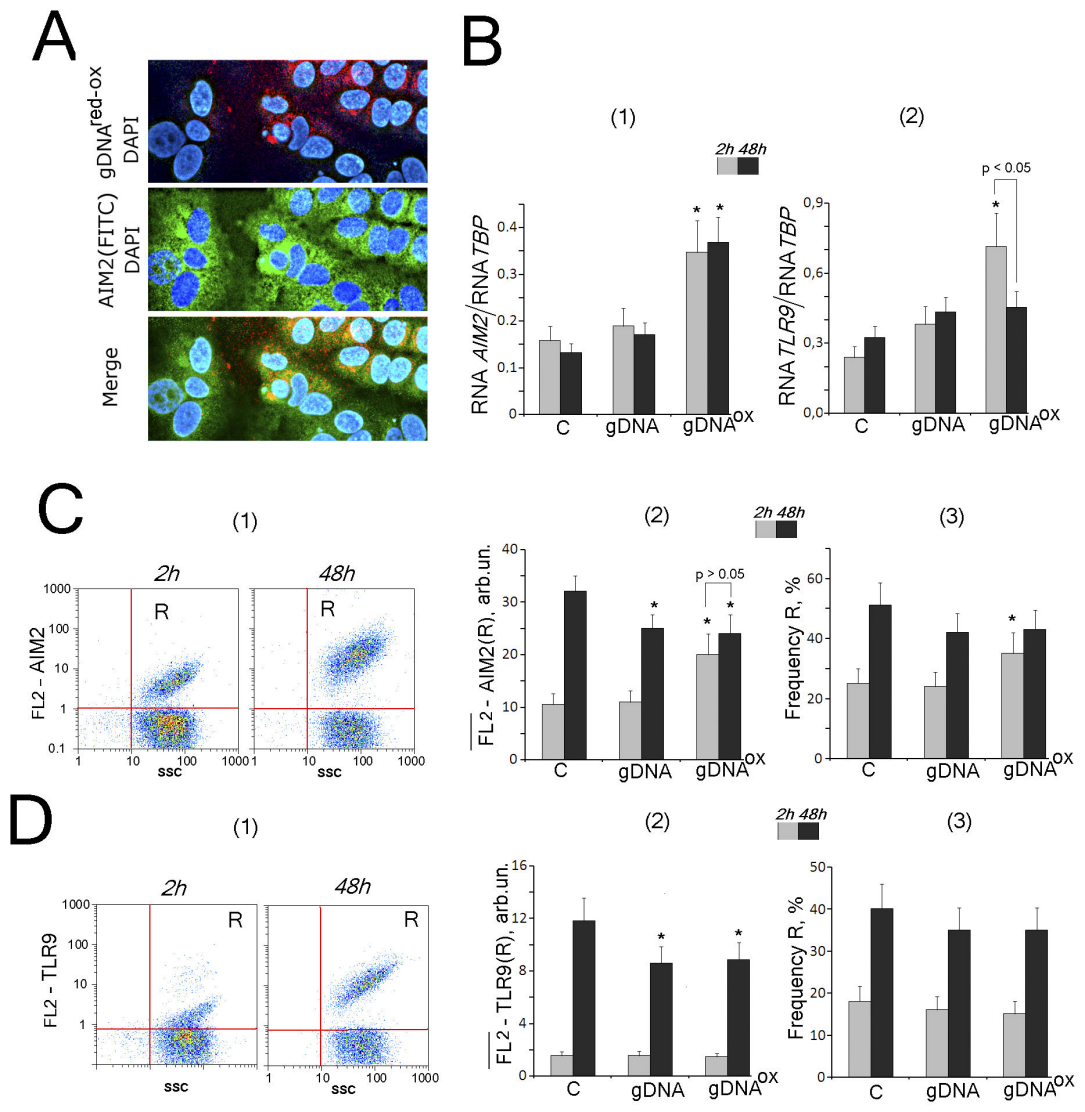
The exposure to gDNA^OX^ (50 ng/mL) leads to a transient increase in expression cytoplasmic DNA sensor AIM2, while not changing expression levels of TLR9. A - intracellular localization of AIM2 (FITC-conjugated antibodies) and labeled probe gDNA**^red-ox^** (x40). B – the ratio of the levels of AIM1 [1] and TLR9 [2] – encoding RNAs to the levels TBP-encoding reference mRNA in cells exposed to gDNA or gDNA^OX^ for 2 hrs (grey columns) and 48 hrs (black columns). C and D – Flow cytometry detection of AIM2 (C) and TLR9 (D) expression in MCF-7. Cells were stained with AIM2 (C) or TLR9 (D) antibody (secondary PE-conjugated antibodies). Panels D [1] and E [1] – control cells plots: FL2 versus SSC. R: gated area. Panels C [2] and D [2]: median signal intensity of FL2 (R) in MCF-7 cells (mean value for three independent experiments). Panels C [3] and D [3]: relative proportions of AIM2- or TLR9-positive cells in R gates [1]. Background fluorescence was quantified using PE-conjugated secondary antibodies. *p < 0.05 against control group of cells, non-parametric U-test.

It is know**n** that intracellular sensors are capable of binding to DNA fragments either inside the cytoplasm (AIM2, RIG1, STING) [27] or within the endosomes (TLR9) [28]. Interestingly, 2-hours exposure to gDNA^OX^ stimulates the expression of mRNAs encoding AIM2, TLR9 and RIG1 (Table 1). Two DNA sensors, AIM2 and TLR9, were studied in greater details (Figure 2).

**Table 1:** The changes in expression levels of select mRNAs after exposure of MCF-7 cells to either gDNA or gDNA^OX^.

**Symbol gene**	**gDNA, 50ng/mL**		**gDNA^OX^, 50ng/Ml**	
	**2h**	**48h**	**2h**	**48h**
**Cell Cycle Checkpoint and Cell Cycle Arrest:**				
***CDKN2A (p16INK4)***	1.8 ± 0.5	3.3 ± 0.3*	1.6 ± 0.1*	2.5 ± 0.3*
***CDKN1A (p21CIP1/WAF1)***	1.3 ± 0.3	2.9 ± 0.2*	1.1 ± 0.2	2.2 ± 0.2*
***TP53***	0.8 ± 0.4	1.6 ± 0.2*	2.6 ± 0.3*	2.1 ± 0.2*
**Anti-Apoptotic**				
***BCL2***	1.2 ± 0.2	2.5 ± 0.3*	3.3 ± 0.3*	3.2 ± 0.2*
***BCL2A1 (Bfl-1/A1)***	1.3 ± 0.3	2.0 ± 0.3*	5.0 ± 0.3*	1.8 ± 0.3*
***BCL2L1 (BCL-X)***	1.0 ± 0.2	1.9 ± 0.3*	1.2 ± 0.3	1.6 ± 0.3*
***BIRC3 (c-IAP1)***	0.7 ± 0.3	3.5 ± 0.4*	1.8 ± 0.2*	2.6 ± 0.4*
**Double Strand Break DNA Repair**				
***BRCA1***	1.0 ± 0.1	1.0 ± 0.1	6.4 ± 0.6*	2.1 ± 0.5*
**Cytoplasmic DNA receptors:**				
***AIM2***	1.2 ± 0.2	1.3 ± 0.1	2.2± 0.2*	2.5 ± 0.4*
***RIG1***	1.5 ± 0.2	1.3 ± 0.2	2.4 ± 0.2*	1.4 ± 0.3
***STING***	1.3 ± 0.2	1.4 ± 0.2	1.0 ± 0.2	1.3 ± 0.3
***TLR9***	1.6 ± 0.2*	1.3 ± 0.2	3.0 ± 0.3*	1.2 ± 0.2
**Nrf2-Keap1 Pathway:**				
***NRF2 (NFE2L2)***	1.4 ± 0.1*	1.1 ± 0.1	2.3 ± 0.1*	1.2 ± 0.2
***KEAP1***	0.9 ± 0.1	1.1 ± 0.1	3.6 ± 0.2*	1.0 ± 0.1
**NFκB Pathway:**				
***MAP4K4***	1.1 ± 0.2	1.5 ± 0.2	2.0 ± 0.1*	1.1 ± 0.3
***MYD88***	1.0 ± 0.2	2.0 ± 0.2*	3.6 ± 0.2*	1.4 ± 0.2
***NFKB1***	1.6 ± 0.2*	0.9 ± 0.2	1.8 ± 0.2*	1.5 ± 0.4
***TIRAP***	1.0 ± 0.2	2.2 ± 0.2*	2.7 ± 0.3*	1.3 ± 0.3
**STAT Family:**				
***STAT3***	1.2 ± 0.2	1.8 ± 0.1*	3.0 ± 0.3*	1.0 ± 0.2
***STAT6***	1.2 ± 0.2	1.8 ± 0.3*	1.6 ± 0.3*	1.1 ± 0.3
**MAPK and JNK/p38 Pathway:**				
***FOS***	1.3 ± 0.2	1.4 ± 0.3	1.4 ± 0.2	1.3 ± 0.3
***JUN***	1.6 ± 0.3*	1.6 ± 0.2*	2.3 ± 0.3*	1.9 ± 0.4*
***MAPK8 (JNK1)***	0.8 ± 0.2	1.8 ± 0.2*	1.3 ± 0.2	1.3 ± 0.2
**Cytokines**				
***IL10***	0.8 ± 0.2	5.3 ± 0.5*	1.8 ± 0.2*	4.2 ± 0.4*
***IL6***	0.8 ± 0.3	1.8 ± 0.2*	2.6 ± 0.3*	1.9 ± 0.2*
***IL8***	1.7 ± 0.2*	1.1 ± 0.2	3.2 ± 0.2*	1.4 ± 0.4
***TNFa***	1.8 ± 0.2*	2.2 ± 0.2*	3.6 ± 0.2*	2.3 ± 0.3*
**Cell Adhesion and Cell Migration Molecules:**				
***ICAM1***	0.9 ± 0.2	1.3 ± 0.2	2.6 ± 0.3*	1.6 ± 0.4
***PECAM1***	1.3 ± 0.2	1.4 ± 0.2	1.7 ± 0.2*	1.2 ± 0.2
***SELE***	1.0 ± 0.1	1.1 ± 0.2	2.1 ± 0.3*	1.0 ± 0.2
***SELP***	3.7 ± 0.3*	1.5 ± 0.2*	1.3 ± 0.2	1.6 ± 0.3*
***VCAM1***	1.5 ± 0.3	1.9 ± 0.2*	3.2 ± 0.3*	1.3 ± 0.2
***RHOA***	1.3 ± 0.2	1.2 ± 0.2	1.6 ± 0.2*	1.1 ± 0.1
**Growth Factors:**				
***BMP2***	1.6 ± 0.2*	1.7 ± 0.2*	3.0 ± 0.3*	2.4 ± 0.2*
***BMP4***	1.2 ± 0.2	1.9 ± 0.3*	2.6 ± 0.4*	1.4 ± 0.4
***VEGFA***	1.3 ± 0.2	1.8 ± 0.4*	0.7 ± 0.3	1.4 ± 0.3
**Pluripotent stem cell-related genes:**				
***NANOG***	1.2 ± 0.3	1.4 ± 0.1*	1.2 ± 0.2	1.0 ± 0.2
***OCT4***	1.2 ± 0.2	1.5 ± 0.2*	2.5 ± 0.2*	1.7 ± 0.1*
***GATA-4***	1.1 ± 0.2	1.5 ± 0.2*	1.4 ± 0.3	1.3 ± 0.3

Relative levels of expression are averages for three biological replicates and a standard deviation. (*) p< 0.05 - against control cells, non-parametric *U*-test (Mann-Whitney U-tests)

#### AIM2

In non-confluent MCF-7 cells, the levels of *AIM2* mRNA (Figure 2B [1]) and protein expression (Figure 2C) are low. In control cells, the protein levels of AIM2 correlate with the degree of confluency. In non-confluent cultures, AIM2 is expressed in about 25 % of cells (Figure 2C [1,3]). In confluent cultures, the proportion of cells with AIM2 increases 2-fold (Figure 2C[1,3]). These increases are paralleled by increases in AIM2 protein levels per cell (Figure 2C[2]), while the levels of AIM2 encoding mRNAs remain approximately the same (Figure 2B[1]). These observations may be explained by prevailing regulation of AIM2 activity at the level of the translation or its stability rather than at the level of transcription and await further investigation.

Merged staining patterns for FITC-conjugated anti-AIM2 antibodies and labeled probe gDNA**^red-ox^** are shown in Figure 2A. Many stained areas, indeed, overlap, possibly indicating an interaction between gDNA^OX^ with AIM2 sensors. In cultured MCF-7 cells exposed to oxidized DNA, the levels of both AIM2 protein and its mRNA are elevated (Figures 2B[1] and 2C). In AIM2-positive population of cells, an exposure to either oxidized DNA or genome DNA for 48 hours leads to the drop in the levels of AIM2 protein per cell (Figure 2С[2]). 

#### TLR9

In non-confluent MCF-7 cells, the levels of TLR9 are low, with approximately 20% of cells stained (Figure 2B[2], D), in agreement with previous studies [28]. In confluent MCF-7 cultures, the proportion of cells expressing TLR9 protein increases to approximately 40% (Figure 2D[3]) along with the intensities of TLR9 staining of individual cells (Figure 2D[2]). Similarly to the levels of AIM2 encodings mRNAs, the levels of TLR9 encodings mRNAs remain unchanged (Figure 2B[2]). After 2 hours of exposure to oxidized DNA, the levels of TLR9 encoding mRNA increase, while amounts of TLR9 protein in individual cells do not change. 

Prolonged exposure of MCF-7 to oxidized DNA leads to a decrease in the intensity of the staining of individual cells with anti-TLR9 antibodies (Figure 2D[2]). Earlier, similar type of the response gDNA and gDNA^OX^ was observed in cultured human fibroblasts [7]. All together, our data indicate that prolonged exposure to either gDNA or gDNA^OX^ leads to the decrease of the cellular levels of DNA sensors AIM2 and TLR9 and, possibly, to partial desensitization of these cells to effects of extracellular DNA. 

### 2. Exposure to gDNA^OX^ induces short-term oxidative stress

**Figure 3:**
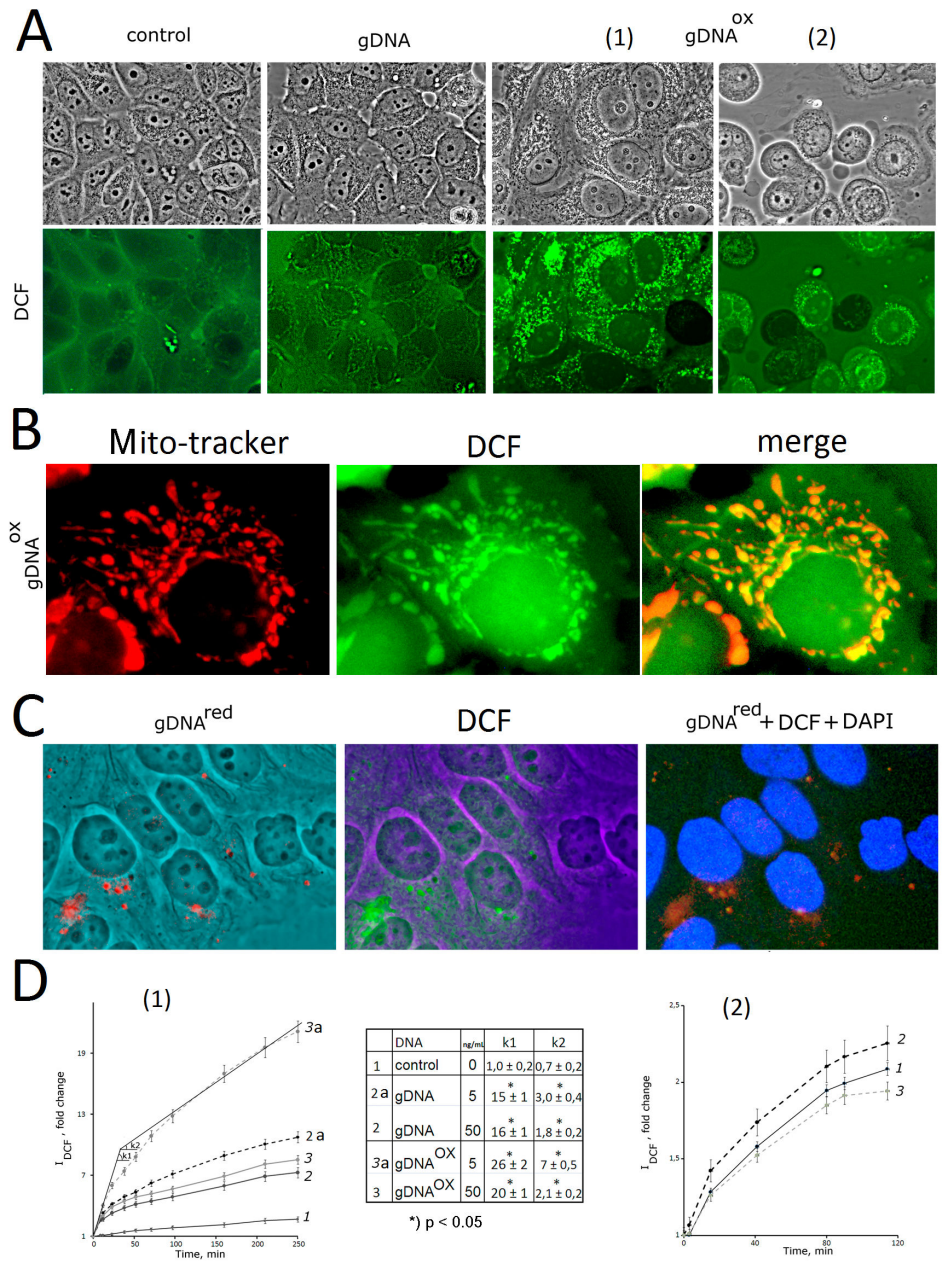
The exposure to gDNA^OX^ leads to an increase in the production of ROS. А – Microscopy-based evaluation of MCF-7 cells sequentially treated with DNA (50 ng/mL) and H2DCFH-DA (control, gDNA, gDNA^ox^ [1]) and incubated for 30 minutes (x100). Alternatively, MCF-7 cells were incubated with DNA (50 ng/mL) for 1 hour followed by addition of H2DCFH-DA and photography 30 minutes later (gDNA^ox^ [2]). B - MCF-7 cells exposed to gDNA^ox^ (0.5h; 50ng/mL), were sequentially treated with Mito-tracker TMRM (15 min) and H2DCFH-DA (15 min) (x200). C - Co-detection of labeled probe gDNA^red^ (50 ng/mL) and DCF after 30 minutes of incubation. D - The results of the quantification of fluorescence using plate reader [1]. The time kinetics of fluorescence outputs in cells sequentially treated with H2DCFH-DA and, three minutes later, a DNA sample at final concentration of 5 or 50 ng/mL [2]. The same for cells pretreated with DNA (final concentration 5 ng/mL) for one hour, with subsequent addition of H2DCFH-DA. *) p < 0.05 against control group of cells, non-parametric U-test.

 To study possible influence of gDNA and gDNA^OX^ on the intracellular levels of reactive oxygen species (ROS), the ROS were measured using dichlorodihydrofluorescindiacetate (H2DCFH-DA) dye that rapidly penetrates cell membranes, and gets trapped in the cytosol in its deacetylated form. Nonfluorescent DCFH transforms to fluorescent DCF by a variety of ROS radicals and, therefore, serves as a sensitive intracellular marker for oxidative stress [29]. Figure 3A depicts the results of the ROS levels analysis in living cells. In untreated control cells, DCF dye diffusely associates with the surface of the cell, and may be removed from the membrane by PBS washing. Most common sources of ROS at cellular membrane are enzymes of NOX family [30]. In cells treated with gDNA (50 ng/mL), H2DCFH-DA stain visualizes both the membrane and some amount of intracellular granules. The PBS wash does not influence cytoplasmic granule staining. Patterns of DCF granules and labeled gDNA**^red^** probe stains approximately overlap (Figure 3C), possibly indicating that an interaction of gDNA with some cellular constituents stimulates ROS biosynthesis at the place of contact. This observation aligns well with previously stated hypothesis that ecDNA may somehow directly stimulate enzymatic activity of NOX proteins [5].

In cells treated with gDNA^OX^ (50 ng/ml), intracellular ROS-producing granules arise fast, and their numbers are substantially larger than in cells treated with gDNA (Figure 3A, inset gDNA^OX^[1]). These events are accompanied by changes in the morphology of MCF-7 cells, including an increase in size of nuclei and cytoplasmic swell. It is important to note that observed cellular responses are rapid and short-living. Described changes in staining patterns and cell morphology are seen only in case of sequential additions of H2DCFH-DA and gDNA^OX^ to MCF-7 media. When cells were pre-treated with gDNA^OX^ for 1 hour, then studied using а H2DCFH-DA dye, the number ROS-synthesizing granules seen in cells was lower and their intensities were less bright than in case of no-pretreatment protocol (Figure 3А inset gDNA^OX^ [2]). Even more interesting, in pre-treatment protocol, some cells stopped ROS biosynthesis at all, and became even less bright then non-treated control cells (darker cells that are less fluorescent than the background (Figure 3А inset gDNA^OX^ (b)).

The observed phenomena were independently confirmed in a study of DCF generation kinetics using quantification with a fluorescent reader (Figure 3D). When MCF-7 cells were treated with DNA immediately after addition of H2DCFH-DA to the media, a dramatic increase in the intensity of DCF fluorescence was observed. These increases were at the highest rates of increase during first 20 minutes after the addition of DNA to the media (coefficient k1), then, with time, these rates drop (coefficient k2) (Figure 3D[1], Table inset). k1 and k2 coefficients were dependent on type and concentrations of DNA treatment: gDNA^OX^ (5ng/mL) > gDNA (5ng/mL) > gDNA^OX^ (50 ng/mL) ≥ gDNA (50 ng/mL) > control. These effects were not seen when cells were pretreated with DNA for 1 hour before the addition of H2DCFH-DA (Figure 3D[2]).

Taken together, the results of these experiments indicate that treatment with gDNA^OX^ rapidly induces ROS biosynthesis in MCF-7 cells. In parallel, the opposite process of the suppression of ROS generation, or ROS quenching, is initiated. As larger the amounts of gDNA^OX^ were added to the media, the more rapid was the development of ROS quenching.

A bulk of the intracellular ROS is generated by mitochondria. An increase in oxidative metabolism in mitochondria may lead to the diffusion of ROS into cytoplasm and subsequent increase in perimitochondrial detection of ROS by DCF. To test this hypothesis, we sequentially stained the cells exposed to 50 ng/mL of gDNA^OX^ for 30 minutes with Mito-tracker (TMRM red) and DCF (Figure 3B). A majority of Mito-tracker and DCF signal were located close to each other, with partially overlaps (yellow signal, Figure 3B). In intact cells, H2DCFH-DA does not stain mitochondria (Figure 3А, control). Our observations point that in the cells exposed to oxidized DNA, a majority of endogenous ROS is generated by mitochondria.

### 3. Exposure togDNA^OX^ stimulates an increase in the levels of oxidative modification of cell’ own DNA

**Figure 4:**
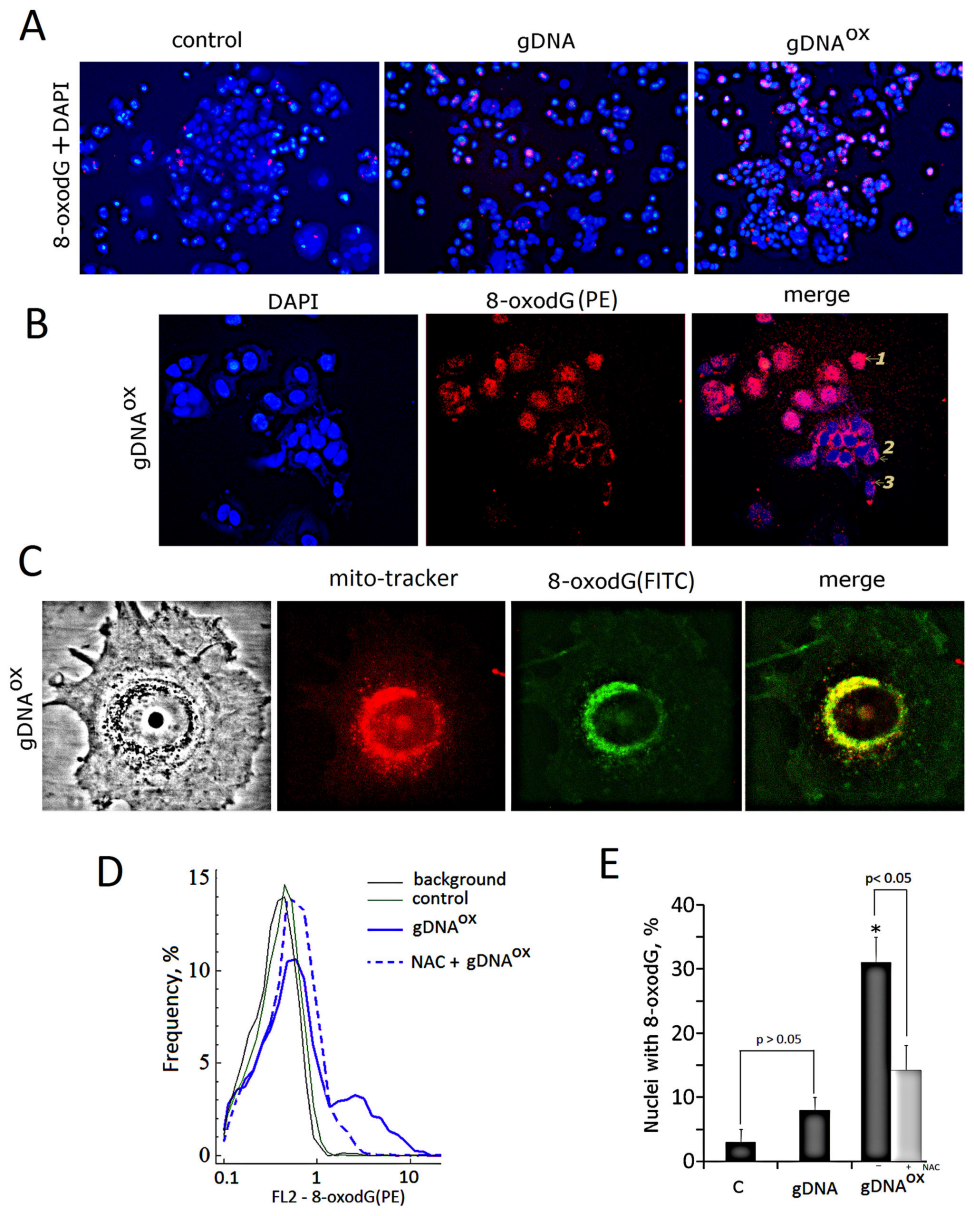
The analysis of 8-oxodG content in cells exposed to either gDNA or gDNA^OX^ (50 ng/mL). A - Cells stained with PE-labeled anti-8-oxodG antibodies and DAPI (x20). B - Three types of anti-8-oxodG stain distribution observed in cells treated with gDNA**^OX^** (x100). Cell were incubated with DNA samples for 1 hour, fixed with 3% formaldehyde, permeated with 0,1 % triton X100 and stained with anti-8-oxodG (PE-conjugated secondary antibodies). C – colocalization of 8-oxodG with mitochondria. Cells were incubated with **gDNA^OX^** for 0.5 hour, обработаны Mito-tracker (30 nM, 15 min), photographed, then fixed with 3% formaldehyde, permeated with 0,1 % triton X100, stained with anti-8-oxodG antibodies (FITC-conjugated secondary antibodies) and photographed again. D - 8-oxodG content in DNA exposed cells pre-treated with NAC (FACS analysis). Cells were incubated with NAC (0.15 mM) for 30 min, then exposed to gDNA^OX^ for 1 hour and analyzed using anti-8-oxodG antibodies (PE-conjugated secondary antibodies). Background fluorescence was quantified using PE-conjugated secondary antibodies. E - Relative proportions of nuclei stained for 8-oxodG in non-treated control cells, cells exposed to gDNA, cells exposed to gDNA^OX^ (grey columns). Light grey column reflects cells pre-treated with NAC and exposed to gDNA^OX^. *p < 0.05 against control group of cells, non-parametric U-test.

It is likely that intensive production of ROS observed immediately after exposure of cells to gDNA^OX^ may result in the damage to cellular DNA. To visualize this damage, fixed MCF-7 cells were stained with PE-labeled anti-8-oxodG antibodies (Figure 4). As compared to non-treated control cells, in MCF-7 cultures treated with either gDNA or gDNA^OX^, the amounts of stained cells were increased (Figure 4A (x20). At larger magnifications, three types of staining patterns may be detected (Figure 4B): (1) – nuclear staining; (2) – cytoplasmic staining; (3) – staining for micronuclei. In non-treated control populations of MCF-7 cells, PE-labeled anti-8-oxodG antibodies predominantly stain micronuclei. In populations treated with gDNA^OX^, there was an increase in the amounts of cells with nuclear staining (Figure 4E). As our previous experiments showed that gDNA^red-OX^ is located in cytoplasm and does not penetrate the nucleus, observed staining of nuclei shall be attributed to the damage of cell’ own DNA. 

An increase of mitochondrial biosynthesis of the ROS in gDNA^OX^ exposed cells demonstrated above (Figure 3В) may lead to an increase in the level of oxidation in mitochondrial DNA that, in turn, may explain observed cytoplasmic staining for gDNA^red-OX^ shown at Figure 1C. On Figure 4C, one may see that some 8-oxodG signals do not merge with gDNA^red-OX^. In cells pretreated with antioxidant N-acetyl-cysteine (NAC) (0.15 mM) for 30 minutes before exposure to gDNA^OX^, the levels of oxidation in cellular DNA were substantially lower than in cells not treated with NAC (Figure 4D and 4E).

### 4. Exposure to gDNA^OX^ stimulates an increase in strand breaks in cell’ own DNA

**Figure 5:**
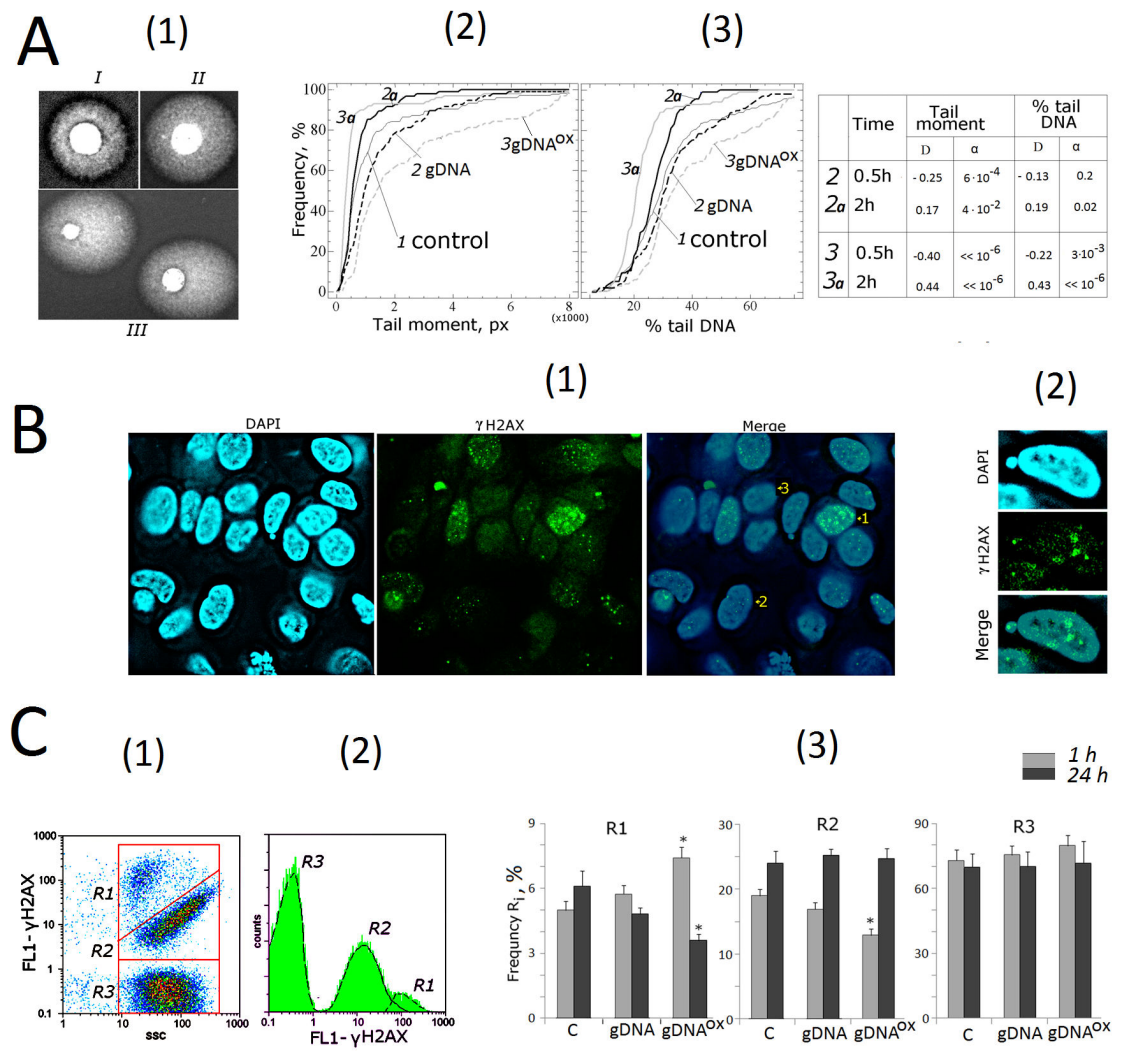
DNA damage in cells exposed to either gDNA or gDNA^OX^ at final concentration 50 ng/mL for 30 min and 2 hours. А – comet assay in alkaline conditions [1]. - Digital photography of the nuclei with varying degree of DNA damage [2,3]; - cumulative histograms for tail moment and percentage of DNA within tails. The reliability of differences with the control in the obtained distributions was analyzed by means of Kolmogorov–Smirnov statistics (the table shows the values of **D** and **α**). B - dsDNA breaks in cells exposed to gDNA**^OX^** (50ng/mL, 1 hour).Cells were processed for immunofluorescence staining with anti γH2AX antibody (x40) [1].- Three detected types of nuclei are denoted by numbers: 1- nucleus with multiple dsDNA breaks, 2- nucleus with a few dsDNA breaks, 3- nucleus with intact DNA [2]. - Example of a micronucleus with dsDNA breaks. С – FACS analysis of γ-foci A: there main fractions of the cells as evident in gating areas R1, R2, R3 [1], the distribution of γH2AX fluorescence intensities [2], relative proportions of cells within gating areas R1-R3 [3]. *p < 0.05 against control group of cells, non-parametric U-test.

One of well-known feature of DNA oxidation is an accumulation of single- and double strand DNA breaks (SSBs and DSBs). To quantify SSBs and DSBs in MCF-7 cells exposed to either gDNA or gDNA^OX^, we employed comet electrophoresis in alkaline conditions (Figure 5А). Three types of nuclei were enumerated: nuclei with intact DNA (Figure 5А [1], Type I); nuclei with some degree of chromatin fragmentation (Type II); nuclei with substantial fragmentation of DNA (Type III). In majority of cases, the nuclei of non-treated control are classified as either Type I or Type II, while Type III nuclei are seen predominantly in cells treated with gDNA^OX^. Depending on how long the cells were exposed to gDNA^OX^, the proportions of Type III nuclei may differ. Figure 5A also presents the comet tail moments [2] and % tail DNA [3]. After 30 minutes of incubation of MCF-7 cells with gDNA^OX^, the amounts of DNA breaks drastically increase, while similar treatment with gDNA leads to moderate elevation of chromatin fragmentation levels. After 2 hours of incubation either with gDNA or gDNA^OX^, the amounts of DNA breaks decrease, and their number falls to below of that found in respective gate-specific populations in non-treated control cells. 

Observations described above were independently confirmed using another common technique for visualization of DSBs, an immunostaining with antibodies against the histone γH2AX, phosphorylated by serine-139. This form of H2AX is known to rapidly accumulate at DNA loci flanking the DSB site [31]. MCF-7 cells stained with FITC-conjugated antibodies to Ser-139 phosphorylated histone γН2АХ are shown at Figure 5B [1]. Stained slides also included three different cell populations of γН2АХ positive cells. In this experiment, cells were classified as Type 1 cells when they had multiple phospho-γН2АХ foci. Most of the γН2АХ positive cells were classified as Type 2 cells (between 2 and 10 distinct γН2АХ foci per cell), and Type 3 cells with no signs of the focal phospho- γН2АХ staining.

In anti-γН2АХ staining, overall fluorescence intensity of the cell is proportional to the number of γН2АХ foci per cell, and, therefore, to amount of DSBs. Using FACS, three gated areas, R1 to R3, were studied (Figure 5C[1,2]). Cells within gate R1 have largest FL1 (γH2AX); this is interpreted as multiple DSBs (Type 1 cells, Figure 5B). Gate R2 contains cells with not numerous γH2AX (Type 2 cells). Gate R3 contains the largest number of cells; most of these cells are intact with no DSBs (Type 3 cells). In MCF-7 cultures, an exposure to gDNA^OX^ (1h) leads to a 1.5-folds increase in the number of cells within gate R1 that is paralleled by a decrease in the number of cells within R2. After 24 hours of exposure to gDNA^OX^, the amounts of cells with multiple DSBs decrease to the levels below that that in non-treated control cells (Figure 5C[3]). A treatment with gDNA evokes similar, but less pronounced type of cellular response that in its magnitude does not reach significance when compared to non-treated control cells (p>0.05). 

These observations indicate that, in MCF-7 cells, short-term exposure to gDNA^OX^ results in both single- and double strand DNA breaks. Longer durations of the treatment (between 2 and 24 hours) evoke some type of compensatory response that leads to a decrease in the levels of chromatin fragmentations across cell populations. 

The drop in the proportion of DSB-containing cells after short-term exposure to oxidized or control DNA may be explained either by the repair of the breaks, or by apoptosis/detachment of damaged cells, or both. To evaluate these possibilities, we enumerated cells that remain in the media after its removal from cell layer, and cells removed from the layer after PBS wash. In cultures exposed to oxidized DNA for 2 hours, the proportion of detached cells remained similar to that in cultures exposed to genomic DNA and non-treated control cultures (approximately 2% of total amount of cells in given culture). Similar results were obtained in experiments aimed at direct evaluation of apoptosis (see below). Therefore, it is likely that the decrease in the proportion of cells with DSBs observed after exposure to gDNA or gDNA^OX^ is due to an increase in DNA repair. 

### 5. Exposure to gDNA^OX^ leads to an increase in genome instability

**Figure 6:**
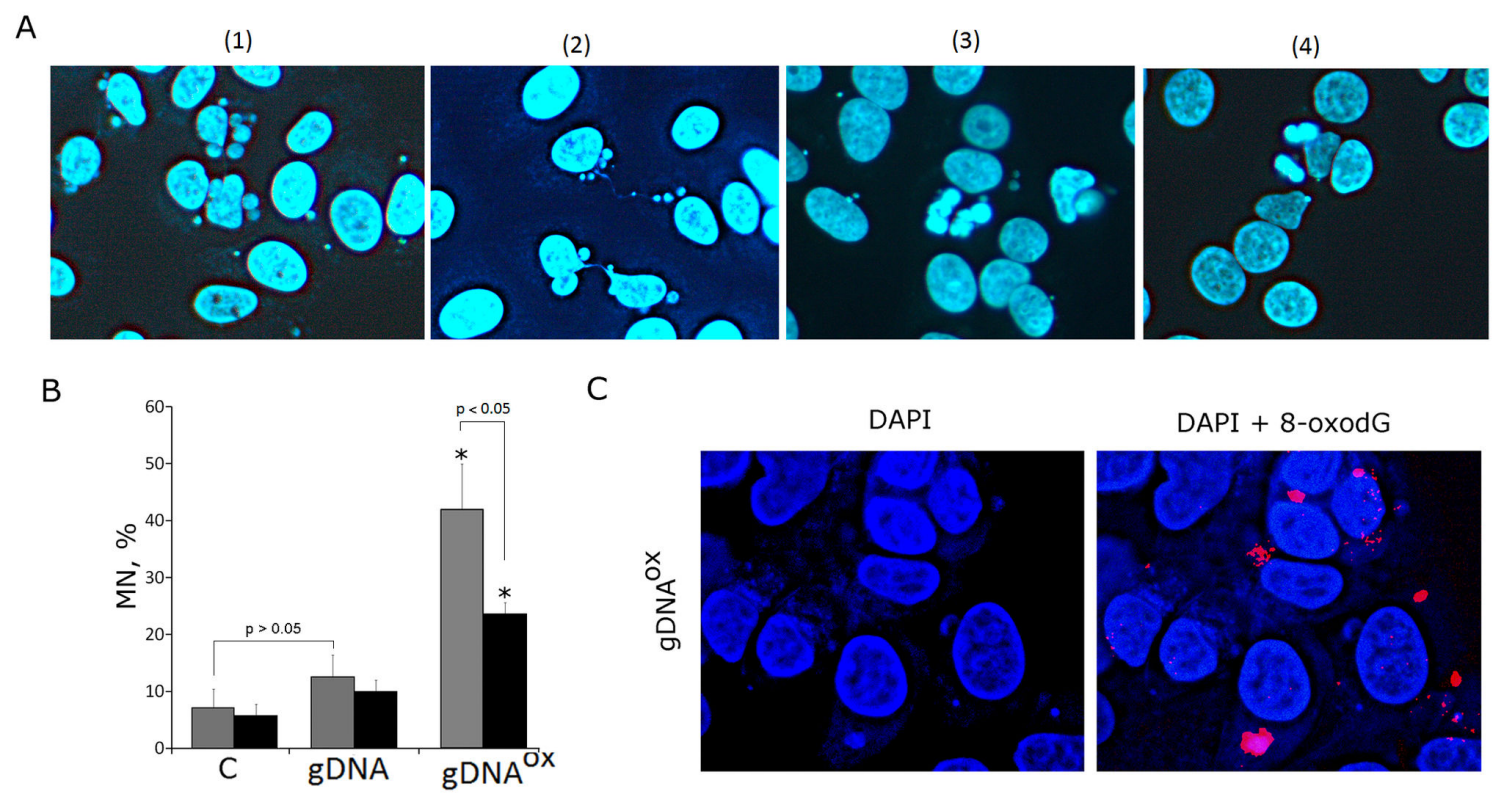
Genome instability in MCF-7 cells exposed to gDNA^OX^ at final concentration 50 ng/mL for 24 hours. A – multiple micronuclei [1], chromatin bridges [2], M-phase chromatin decondensation [3], non-treated control cells [4] (x100). B – proportions of cells with micronuclei in non-treated control cells, cells exposed to gDNA, cells exposed to gDNA**^OX^**. Grey columns: non-confluent, actively proliferating MCF-7 culture. Black columns: MCF-7 cells at high confluency. *p < 0.05 against control group of cells, non-parametric U-test. С - Exposure to gDNA**^OX^** (50 ng/mL, 2 hours) induces formation of 8-oxodG-containing micronuclei (x100).

Single- and double strand DNA breaks are known to result in the loss of chromosome stability that is especially prominent in actively proliferating cells [32]. A thorough study of the nuclei of the cells incubated with gDNA^OX^ revealed pronounced chromosome instability (Figure 6). At concentrations of 50 ng/mL, an exposure of actively proliferating, low confluency MCF-7 cells to gDNA^OX^ results in the formation of multiple micronuclei (Figure 6A[1]) and other nuclear anomalies such as nucleoplasmic bridges and nuclear buds (Figure 6A[2]), as well as in decondensation of mitotic chromosomes (Figure 6A[3]). All of these events are signs of profound replication stress that is known to develop in actively proliferation cell cultures undergoing various stress treatments [32]. Similarly treated cell cultures with lower proportions of proliferating cells, for example, confluent or serum starved cultures show substantially lesser the amounts of chromatin changes. Proportions of micronuclei-containing cells in cultures grown in varying conditions are show at Figure 6В. In non-treated control MCF-7 cells, the frequency of cells with micronuclei was around 7%, a number that is similar to that reported in other studies [33]. In actively proliferating cultures exposed to gDNA^OX^, the micronuclei were detected in about 40% of cells. Exposure to gDNA also leads to increase in the amounts of cells with micronuclei, but in this case an increase is not significant. Many micronuclei formed after the treatment with gDNA^OX^ were positively stained for both PE-labeled anti-8-oxodG (Figure 4B and Figure 6C) and anti-phospho-γН2АХ antibodies that highlight DSBs (Figure 5B [2]). 

These observations indicate that, in MCF-7 cells, an exposure to gDNA^OX^ induces genome instability that is, most likely, secondary to accumulation of large the amounts of SSBs and DSBs.

### 6. Exposure to gDNA^OX^ arrests cell cycle

**Figure 7:**
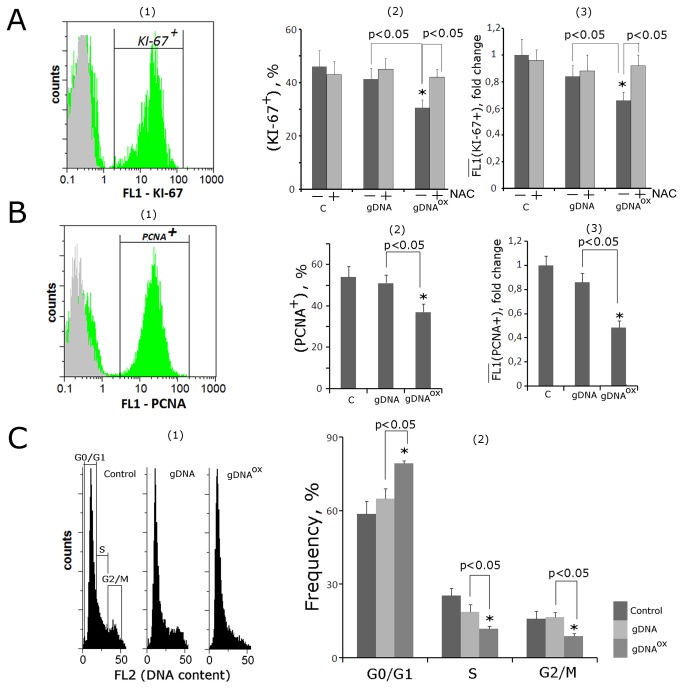
Proliferation and cell cycle of MCF-7 cells exposed to gDNA or gDNA^OX^ at final concentration 50 ng/mL for 48 hours (FACS). A: (1) - fixed cells stained with anti-Ki-67 antibodies (green color). Background fluorescence was quantified using FITC-conjugated secondary antibodies (grey color) [2]. - proportion of Ki-67-positive cells in total cell population [3]. - the average signal intensity of FL1 (Ki-67+). Cells were cultivated either in absence (dark grey columns) or in presence of 0.15 mM NAC (light grey columns). B: (1) - fixed cells stained with anti-PCNA antibodies (green color). Background fluorescence was quantified using FITC-conjugated secondary antibodies (grey color) [2]. - proportion of PCNA-positive cells in total cell population [3]. - the average of the median signal intensities of FL1 (PCNA+). C: (1) - distribution of fluorescence intensities of the cells stained with пропидий йодидом. (2) - содержание в популяции клеток с количеством ДНК, соответствующим G1-, S and G2/M –фазам клеточного цикла. *p < 0.05 against control group of cells, non-parametric U-test.

One of the most important consequences of genome instability is the block of cell proliferation due to activation of the DNA damage checkpoints. Cell cycle-related consequences of exposure to or gDNA were studied in MCF-7 cells that were harvested 48 hours after addition of DNA (50 ng/mL) to the media (Figure 7). 

To investigate these cultures, cells were stained with antibodies to the proliferation markers Ki-67 and PCNA [34,35] and enumerated by FACS. Additionally, cell counts were also performed after DNA-specific propidium iodide (PI) treatment. Figure 7A shows the distribution of the cells with various Ki-67 contents. In control MCF-7 cultures, Ki-67 stains approximately 45% of cells. After exposure to gDNA^OX^, the proportion of Ki-67-positive cells decreased to 30% (Figure 7A[2]). These decreases were paralleled by the decrease in mean fluorescence intensity per each Ki-67-positive cell by 40% that is indicative of the decrease in amounts of Ki-67 in individual cells. Similar results were obtained using another well-known marker of proliferation, PCNA (Figure 7B[1-3]). It seems that observed block of proliferation is ROS-dependent, as the changes in KI-67 staining of the cells pre-treated with antioxidant NAC (0.15mM) and exposed to same amounts of oxidized DNA were not significant (Figure 7С[2,3]).

The data collected after the staining with propidium iodide (PI) point to similar direction (Figure 7С[1]). After exposure to gDNA^OX^, the proportion of G0/G1 cells increased, while proportions of the cells in S- and G2/M phases decreased (Figure 7С[2]). These observations indicate that, in a substantial proportion of previously proliferating MCF-7 cells, the exposure to gDNA^OX^ and, to a lesser degree, to gDNA, blocks the cell cycle in G0/G1.

This line of evidence was also supported by qRT-PCR analysis at the level of mRNA encoding inducible cell cycle arrest proteins, including CDKN2A (p16INK4), CDKN1A (p21CIP1/WAF1) and TP53 (Table 1). Cell cycle changes evoked by treatment with gDNA were similar to those of gDNA^OX^, but substantially less pronounced.

### 7. Exposure to either gDNA^OX^ or gDNA supports cell survival

**Figure 8:**
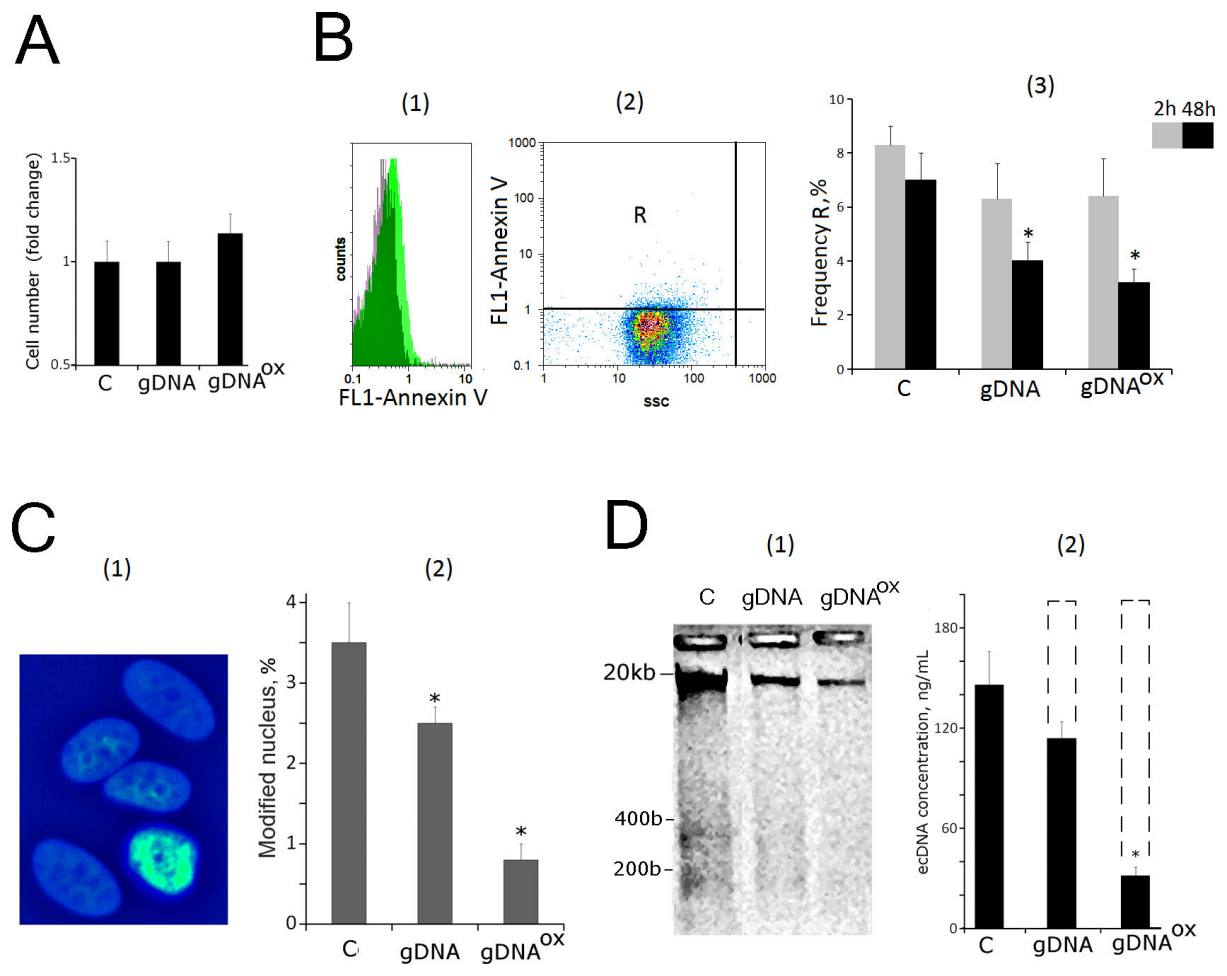
Cell death in MCF-7 cultures exposed to either gDNA or gDNA^OX^ at final concentration 50 ng/mL for 48 hours. A. Total number of cells in studied cell population. B. (FACS) – enumeration of cells with sings of early apoptosis [1]. - the distribution of fluorescence intensities of the cells stained with Annexin V-FITC (green color) или FITC-conjugated secondary antibodies (grey color) [2]. - control cells plots: FL1 versus SSC. R: gated area [3]. - the proportion of Annexin V -positive cells in total cell population. C. Evaluation of modified nuclei in three studies typed of MCF-7 cultures. (1) -Example of Hoechst33342 staining; (2) - Graph of the proportion of cells with modified nuclei in three studied types of MCF-7 cultures. D. Electrophoresis [1] and evaluation of ecDNA concentrations [2] in the media of non-treated control cells and cells exposed to either gDNA or gDNA^OX^. Dashed line indicates amounts of ecDNA that should be present in the media when exogenous DNA is taken into account. *p < 0.05 against control group of cells, non-parametric U-test.

It was noted that the total amount of cells harvested 48 hours after exposure to gDNA^OX^ or gDNA were similar to those of non-treated control populations (Figure 8А). As the proliferation activities of cells treated with either gDNA^OX^ or gDNA were, at least in part, blocked (Figure 7), it was important to evaluate overall levels of cell death in all studied populations. 

To quantify cells in early apoptosis, we used FITC-conjugated Annexin V (Figure 8B[1-3]). After two hours of exposure either gDNA^OX^ or gDNA, the proportion of the apoptotic cells went down approximately by 25%, but observed changes had not reached significance (p>0.05)). However, after 48 hours of exposure to either gDNA^OX^ or gDNA, the proportion of apoptotic cells in treated cultures decreased to the levels twice less than in control MCF-7 cultures. 

To evaluate overall levels of cell death in all studied populations, nuclear morphology was evaluated in all populations after staining with Hoechst33342 (Figure 8C [1,2]). If condensed and fragmented chromatin was detected, the cell was marked as apoptotic. After exposure to gDNA^OX^ (48 hours, 50 ng/mL), the amount of cells with apoptotic nuclei decreased three folds. 

To further assess various aspects of cell death, we extracted ecDNA from cell-free media conditioned by non-treated control cells and cells treated either with gDNA or gDNA^OX^ for 48 hours (50 ng/mL). Extracted DNA fragments were analyzed by gel electrophoresis to assess their size distribution (Figure 8D[1]). The length of DNA fragments extracted from cell-free media conditioned by non-treated control cells, varied between 15 kb and 0.1 kb, and included visible mono- and dinucleosome bands that are contributed to the ecDNA pool by dying apoptotic cells [36]. In cells treated either with gDNA or gDNA^OX^, these bands were less prominent. The decrease in relative abundance of mono- and dinucleosome bands was in concert with the overall decrease in total amounts of ecDNA extracted from cell-free media and quantified using RiboGreen stain (Figure 8D[2]). In media of MCF-7 cells exposed to exogenous DNA, the final concentrations of ecDNA should be around 190 ng/mL (a sum of concentrations of endogenously produced DNA at 140 ng/mL and added DNA at 50 ng/mL); However, cell-free media of cells treated with exogenous DNA had substantially lower concentrations of DNA, in fact, after treatment with gDNA, these concentrations were 1.7 times lower than expected. After treatment with gDNA^OX^, these concentrations were 6 times lower than expected. These drastic drops in DNA concentrations may be explained by the decrease of overall levels of apoptosis and DNA release in gDNA or gDNA^OX^ treated cultures.

Figure 8 presents evidence that in gDNA^OX^ treated MCF-7 cultures and, to lesser degree, in gDNA treated cells, the levels of cell death substantially decrease as compared to non-treated controls. Additional supportive evidence for this statement is presented in Table 1 that summarizes the changes in expression levels for mRNAs encoding cell survival and DNA repair related proteins. In two hours after adding gDNA^OX^ to MCF-7 culture, levels of mRNA for *BCL2, BCL2A1* (Bfl-1/A1)*, BCL2L1* (BCL-X)*, BIRC3* (c-IAP1) and *BRCA1* increase 1.2 to 6.4 folds, and stay elevated for at least 48 hours. In case of treatment with gDNA, these genes also tend to increase their mRNA biosynthesis, up to 1.9 - 3.5 times, but these changes in expression levels are delayed as compared to the treatment with gDNA^OX^ and reach significance only after 48 hours. Interestingly, in case of treatment with gDNA, the expression levels of mRNA encoding for key component of DSB repair machinery *BRCA1*, were not altered.

### 8.  Exposure to either gDNA^OX^ or gDNA leads to a decrease in activity of NRF2 and an increase in activity of NF-kB and STAT3

**Figure 9:**
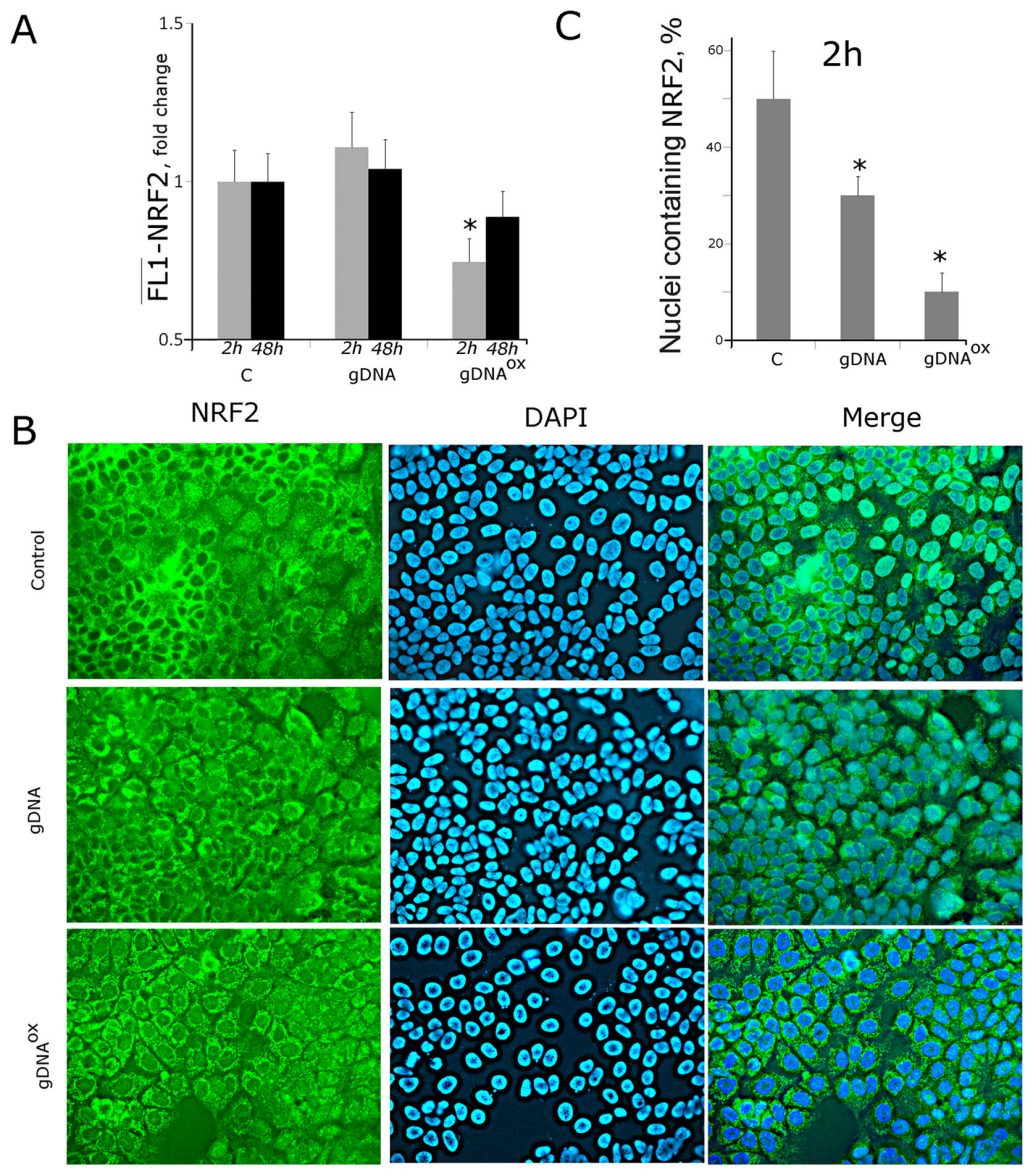
Decrease in activity of transcriptional factor NRF2 in MCF-7 cells exposed to gDNA^OX^ at final concentrations of 50 ng/mL for 2 hours. A FACS: the average of the median signal intensities in cells stained with anti-NRF2 antibodies after various exposures. B - Fluorescent microscopy of cells stained to NRF2 (x40). C - Graph of the proportion of cells with nuclear staining for NRF2 in three studied types of MCF-7 cultures. *p < 0.05 against control group of cells, non-parametric U-test.

NF-E2-related factor 2 (NRF2) is known to participate in the development of adaptive response in fibroblasts and mesenchymal stem cells cultivated in the presence of gDNA^OX^ [5,7]. After 2 hours of exposure of MCF-7 cells to gDNA^OX^, the levels of *NRF2* mRNA increase (Table 1). At the same time point, there is an increase in the expression of the gene *KEAP1* that encodes for a cytoplasmic protein partner of NRF2, capable of blocking its transcription factor activity [37]. As evident from FACS data, protein levels of NRF2 after treatment with gDNA do not change (Figure 9A). An exposure to **gDNA^OX^** for 2 hours leads **to a decrease of** NRF2 levels. Fluorescent microscopy studies showed that exposure to gDNA^OX^ leads to a change in the NRF2 staining pattern. In non-treated control MCF-7 cells, NRF2 is located both in the nucleus (^~^50% of cells) and in the cytoplasm (most of the cells), while in cells exposed to gDNA^OX^ NFR2 is found exclusively in the cytoplasm (Figure 9B), thus, indicating suggesting that its transcriptional activator function is blocked.

NF-κB and STAT3 control the expression of anti-apoptotic and cell cycle control and proliferation genes. Both of these transcriptional factors are activated in response to various kinds of stress. In particular, NF-κB and STAT3 were found to play pivotal roles in various aspects of tumorigenesis [38,39]. Here we present an analysis of activity of these two transcription factors in cells exposed to either gDNA or gDNA^OX^.

#### NF-κB

**Figure 10:**
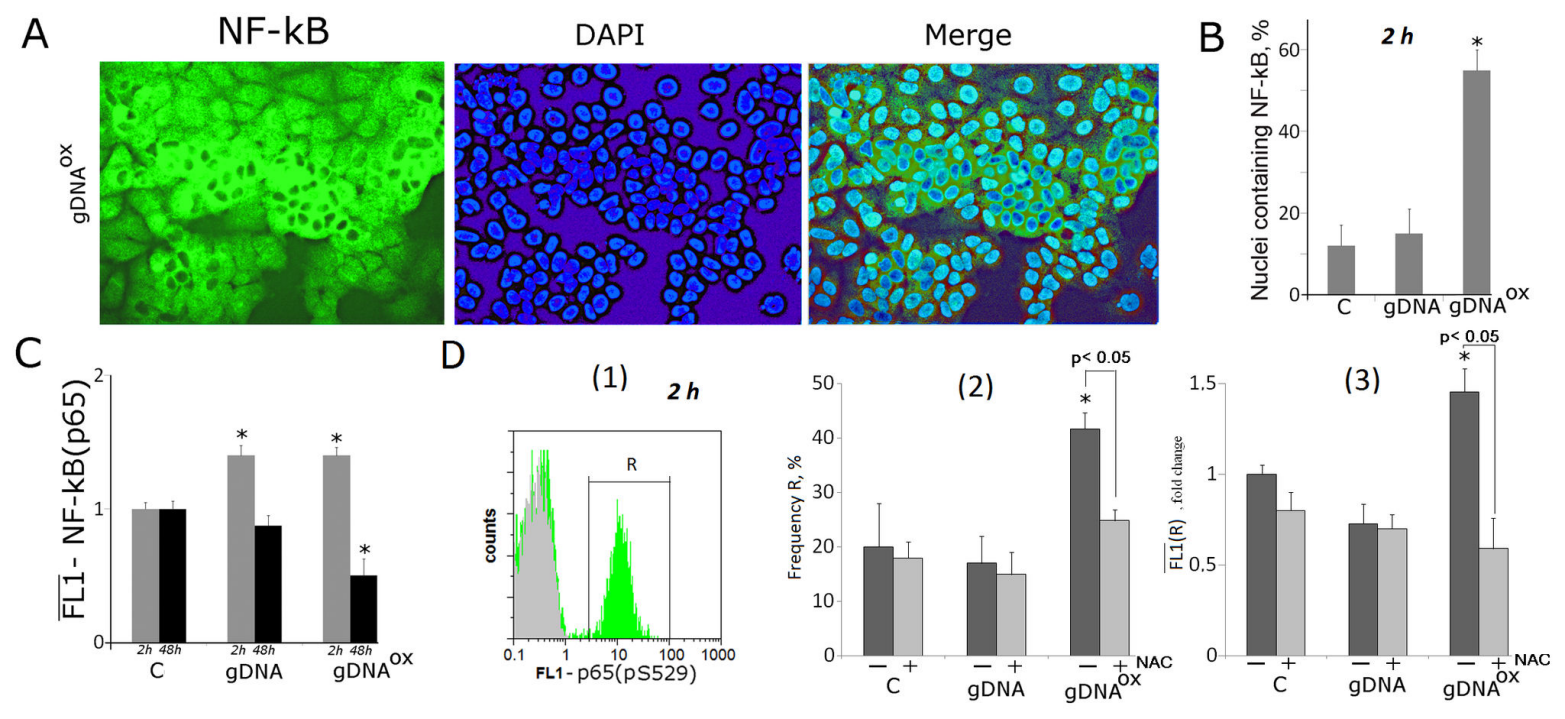
Increase in activity of transcriptional factor Nf-kB in MCF-7 cells exposed to gDNA^OX^ at final concentrations of 50 ng/mL for 2 hours. A Fluorescent microscopy of cells stained with anti-p65 (FITC) antibodies (x40). B Graph of the proportion of cells with nuclear staining for Nf-kB in three studied types of MCF-7 cultures. C, D (FACS) - the average signal intensity of FL1 (p65) in cells stained with anti-p65 (C) and Ser529-phosphorylated р65 (D) antibodies [1]. - distribution of fluorescence intensities of the cells stained with Ser529-phosphorylated р65 antibodies (FITC) (green color) или FITC-conjugated secondary antibodies (grey color) [2]. - proportion of Ser529-phosphorylated р65 -positive cells in total cell population [3]. - the average of the median signal intensities of FL1 (Ser529-phosphorylated р65 +). Cells were cultivated either in absence (dark grey columns) or in presence of 0.15 mM NAC (light grey columns).

The exposure to gDNA^OX^ leads to a rapid, 1.8-3.6 fold increase in the levels of mRNAs encoding components of the NF-κB pathway, including *MAP4K4, MYD88, NFKB1* and *TIRAP* (Table 1).The effects of exposure to gDNA are seen substantially later, at 48 hours post exposure (*MAP4K4, MYD88* and *TIRAP*). After 2 hours of exposure to either gDNA or gDNA^OX^, the amount of NF-κB (p65) proteins increase 1.5 fold (FACS, Figure 10C), and decrease 48 hours later. Fluorescent microscopy evaluation of gDNA^OX^-treated MCF-7 cells confirms activation of NF-κB as evident from the translocation of this factor into the nucleus (Figure 10A). After 2 hours of exposure, the fraction of MCF-7 cells with nuclear staining for NF-κB increases from 12% to 56% (Figure 10B). 

 It is known that NF-κB (р65) is activated by phosphorylation, which plays a key role in the regulation of its transcriptional activity and is associated with nuclear translocation. For instance, upon treatment with TNFa, Ser529 of р65 is phosphorylated by casein kinase II [40]. Flow cytometry quantification (Figure 10D) demonstrates that exposure to gDNA^OX^ leads to an increase of the proportion of cells that contain Ser529-phosphorylated р65, thus, confirming that NF-κB in these cells is transcriptionally active [40]. The exposure to gDNA does not increase the proportion of cells with Ser529-phosphorylated р65. The pre-treatment with antioxidant NAC at 0.15mM for 30 minutes before addition of same amount of oxidized DNA prevented an increase in the levels of Ser529-phosphorylated р65 that remained similar to that in control cells (Figure 10D [2,3]). Therefore, we may conclude that oxidized DNA dependent activation of NF-κB is mediated by an increase in local production of ROS. 

#### STAT3

**Figure 11:**
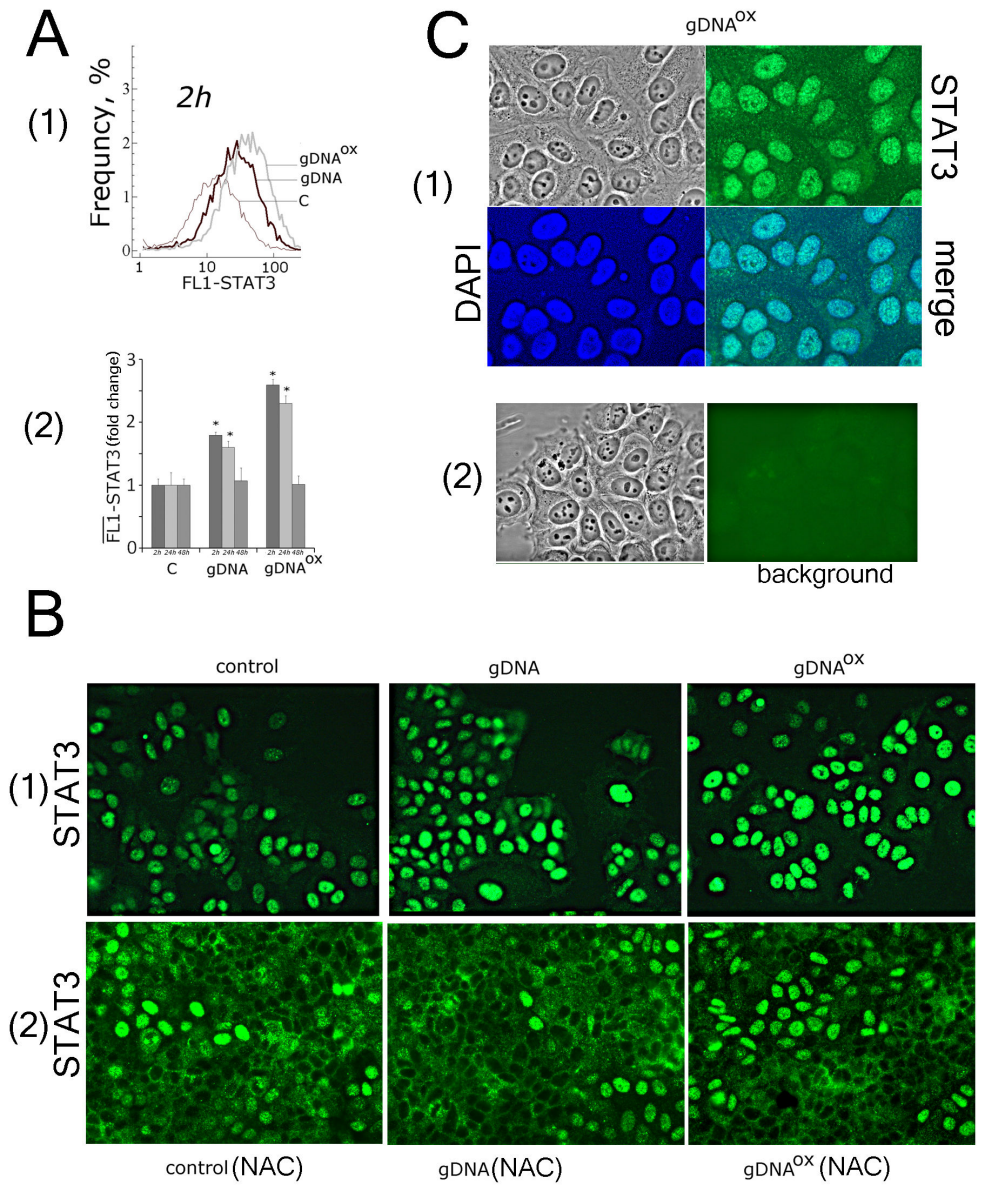
Activity of STAT3 is stimulated in MCF-7 cells exposed to either gDNA or gDNA^OX^ at final concentrations of 50 ng/mL. A FACS: Frequency plot for fluorescence intensities in cells stained with anti-STAT3 antibodies [1] and the average of the median signal intensities of FL1 (STAT3) in these cells [2]. B Fluorescent microscopy of cells stained with STAT3 antibodies (x20) [1]. - non-treated control cells and cells exposed to either gDNA or gDNA**^OX^** for 2 hours [2]. - cells pre-treated for 30 min by 0.15mM NAC, then exposed to either gDNA or gDNA**^OX^** for 2 hours. С [1] - evidence for nuclear localization of STAT3 (x100), the nuclei were stained with DAPI [2]. - to evaluate the background, the cells were treated with normal rabbit IgG and FITC-conjugated secondary antibodies.

Two hours exposure to gDNA^OX^ also leads to an increase in the expression of mRNA for *STAT3* and *STAT6* (3 and 1.6 fold, respectively) (Table 1), while exposure to gDNA results in significant activation of *STAT3* and *STAT6* only at the 48 hour time point. Both FACS and fluorescent microscopy show that non-treated control MCF-7 cells express substantial amounts of STAT3 (Figure 11A[1,2], 11B[1] и 11С). Importantly, in these cells STAT3 is located exclusively in the nuclei. These observations indicate that STAT3 in active in control MCF-7 cultures. Published studies describing activity of Stat3 in MCF-7 contradict each other. Some authors showed that in MCF-7 Stat3 is phosphorylated and located in the nuclei [41]. Other studies failed to detect activity of Stat3 in MCF-7 [42]. Stat3 activity may change in response to growth factors and cytokines [38,39]. Therefore, observed disagreements may be explained by differing cultivation conditions, in particular, by type of the serum supplementation. Interestingly, supplementation of the media with antioxidant NAC leads to decrease in activity of Stat3 (Figure 11B[2]).

After 2 hours of exposure to either gDNA^OX^ or gDNA, the amounts of STAT3 increase, with no changes in its localization. In 24 hours, the amounts of STAT3 protein start to decrease and in 48 hours after the addition of DNA, samples reach their initial levels (Figure 11A[2]). In the case of exposure to gDNA^OX^, these effects are more pronounced than in the case of gDNA. The pre-treatment with antioxidant NAC at 0.15mM for 30 minutes before addition of same amount of oxidized DNA prevented activation of STAT3.

Both gDNA^OX^- and gDNA-induced activation of NF-κB and STAT3 leads to an increase in the expression levels of genes encoding components of MAPK and JNK/p38 pathway: *FOS, JUN* and *MAPK8* (*JNK1*). In parallel, we observed an increase in the expression of genes that encode soluble cytokines (Table 1). For *IL10, IL6, IL8* and *TNFa*, the levels of mRNA increase 1.8-5.3 folds; two hours after adding DNA sample to the media, in gDNA^OX^- treated MCF-7 cells, the levels of these mRNAs are 2-3 times higher than those in cells treated with gDNA. Additionally, we observed expression stimulating effects of gDNA^OX^ on cell adhesion and migration molecules *ICAM1, PECAM1, SELE, SELP, VCAM1*,and *RHOA, g*rowth factor encoding genes *VEGFA, BMP4* and *BMP2* and pluripotent stem cell-related genes *NANOG, OCT4* and *GATA-4* (Table 1).

## Discussion

High levels of cell-free DNA were found in cancer patients and in relevant in vivo models previously [43]. Moreover, substantially larger degrees of cfDNA fragmentation were observed both in cancer patients and in nude mice xenograft models, pointing to apoptotic cells as a possible source of cfDNA [44]. It is likely that the DNA released from dying cells as a result of oxidative insult, i.e. irradiation or chemotherapy-associated oxidative stress, is also damaged. Thus, all over the body, cells experience both an increase in the quantities of extracellular DNA and have increased proportion of damaged/unusual nucleotide bases within extracellular DNA fragments.

In this study we attempted to model an event that is naturally occurring in the body of patients exposed to cell death-inducing antitumoral therapy, an increase in the level of damaged, circulating DNA released from dying cells. As the model cell line, we selected the estrogen-sensitive breast adenocarcinoma cell line MCF-7 that is particularly well characterized and widely accepted for cancer studies.  Media conditioned by MCF-7 cells contains substantially larger amounts of extracellular DNA (140 ng/mL) as compared to a variety of normal cells that we profiled previously, including fibroblasts [7], endotheliocytes [15] and mesenchymal stem cells [5,6] (6 -30 ng/mL).  

One of the most important conclusions of our study is that normal, non-oxidized extracellular DNA penetrates the cells, but remains at the cytoplasmic foci close to the membrane. The number of these foci depends on the properties of extracellular DNA, in particular, on the degree of its enrichment in guanine and cytosine. It is likely that the binding of extracellular DNA to the cell membrane is mediated by receptors with varying affinities to different DNA sequences. It is also possible that the kinetics of ecDNA binding to the surface of MCF-7 cells differ from that of normal cells, due to larger concentrations of ecDNA in the media.

**Figure 12:**
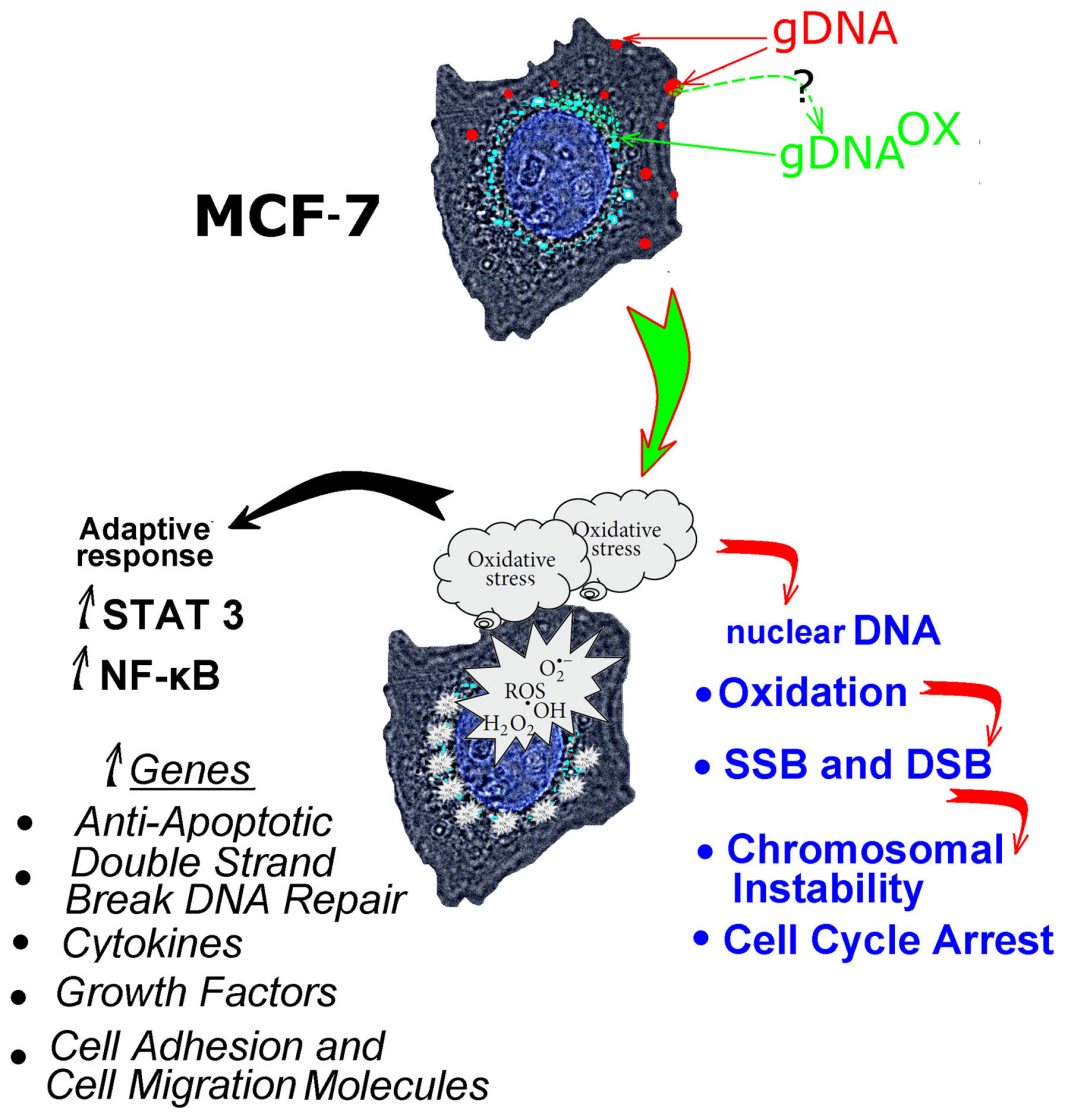
A summary of events developing in MCF-7 cells exposed to oxidized DNA, and possible mediators of an adaptive response observed in these cells.

Intracellular distributions of oxidized and regular genomic DNA differ (Figure 12). The fragments of gDNA^OX^ are located closer to the nucleus than similarly prepared fragments of regular gDNA (Figure 1 A-C). An increase in expression of early endosomal marker EEA1 indicates that most likely mechanism for gDNA^OX^ penetration into the cells is through endocytosis (Figure 1D). Some fraction of non-oxidized genomic DNA is also found at perinuclear locations (Figure 1B); this is possibly due to secondary oxidation of DNA at the points of focal contact with the cell surface [5]. This hypothesis is supported by the local activation of ROS biosynthesis at DNA-associated foci (Figure 3С). After oxidation, genomic DNA may be delivered inside the cell through the same pathway as gDNA^OX^ (Figure 12).

After delivery into the cytoplasm, gDNA^OX^ immediately induces the burst of ROS (Figure 3A). So far, we do not know much about the particular mechanism that connects gDNA^OX^ to ROS-generating cascades. However, our data indicate that gDNAOX induces the production of ROS by mitochondria (Figure 3B). 

The perinuclear production of ROS leads to either the direct damage to the genomic DNA of affected cells or to the increase in nuclear pool of free 8-oxodG that may affect genomic DNA of the cell through its salvage and incorporation into DNA [45,46]. In any case, exposure to gDNA^OX^ leads to an increase of 8-oxodG content in mitochondrial DNA (Figure 4С), in the nuclear staining for 8-oxodG (Figure 4) and the amounts of SSBs and DSBs in cell’ DNA (Figure 5). In turn, the accumulation of DNA breaks blocks cell proliferation through activation of checkpoints (Figure 7). In addition, we observed an increase in other signs of genome instability, in particular, the number of micronuclei and other nuclear anomalies such as nucleoplasmic bridges and nuclear buds (Figure 6). Therefore, the overall trend of MCF-7 cells response to exposure to gDNA^OX^ is an increase in the levels of damage to the cell’ own DNA followed by the block of the division, and possibly, activation of DNA repair machinery. 

Importantly, a burst in ROS biosynthesis that is observed in the first 30 minutes after adding gDNA^OX^ to the media is accompanied by an increase in anti-oxidant responses. After an hour of MCF-7 incubation with gDNA^OX^, the levels of ROS biosynthesis drop below those seen in control, non-exposed cells (Figure 3). Interestingly, the antioxidant responses of MCF-7 cells do not depend on activity of NRF2, a basic leucine zipper redox-sensitive transcriptional factor that plays a center role in ARE (antioxidant response element)-mediated induction of phase II detoxifying and antioxidant enzymes. In non-cancerous cells treated with gDNA^OX^, NRF2 mediates a set of adaptive responses [5,7]. Moreover, in MCF-7, NRF2 remains inactive despite nuclear translocation of oxidant-sensitive transcription factor NF-kB that controls expression of genes involved in immune and inflammatory responses. Crosstalk between NRF2 and NF-κB is an area of extensive interest. Typically, activation of NRF2 is accompanied by the block of NF-κB signaling pathways, and *vice versa* [47,48]. Exposure to gDNA^OX^ leads to activation of NF-κB, evident from an increase in mRNA levels for the components of NF-κB signaling pathway, elevation in the levels of p65 and its active, phosphorylated isoform as well as the nuclear translocation of p65, observed in 60% of cells (Figure 10). In addition to the activation of NF-κB, exposure to gDNA^OX^ results in the upregulation of STAT3, known to promote the development and progression of some types of cancers [38,39]. After exposure of MCF-7 cells to gDNA^OX^, the levels of both *STAT3* mRNA and its protein increase approximately 2.5 folds [Figure 11]. Interestingly, the transcription factor STAT3 has recently been found to suppress mtROS production independent of its nuclear factor activity [49].

Concerted activation of NF-kB and STAT3 is followed by an increase in expression levels of genes associated with cell survival. After 48 hours of exposure to gDNA^OX^, a decrease in MCF-7 cell death was observed. These effects were seen not with standing an initial burst in ROS biosynthesis and extensive DNA damage observed in the beginning of the treatment with oxidized DNA. In gDNA^OX^-treated cultures, a decrease in cell proliferation is paralleled by a decrease in cell death events, reflected by the lack of net change in the total amounts of cells in the culture wells (Figure 8). 

It seems that the effects of oxidized DNA are, at least in part, mediated by transient increase in the perimitochondrial levels of ROS. This is evident from experiments with experiments on cells pretreated with antioxidant NAC that precludes or substantially decreases the magnitude of gDNA^OX^-dependent effects, in particular, the genomic DNA oxidation (Figure 4 D,E), the block of the cell cycle (Figure 7) as well as the activation of NF-kB (Figure 10) and STAT3 (Figure 11). 

Taken together, our study indicates that exposure to oxidized DNA increases survivability of the tumor cells. These effects have substantial therapeutic relevance, as typical antitumoral therapy leads to massive cell death that, in many instances, includes a substantial oxidative damage related component [50], and, therefore, contributes to the release of oxidized DNA. Additionally, even in untreated tumors, the high endogenous levels of reactive oxygen species [51,52] results in increased levels of apoptosis that, in turn, increases the amounts of oxidized DNA that, in turn, leads to a homeostatic return to balance through stimulated increase in cell survival. This logic is consistent with the findings of Iwasa Y et al., that high rates of apoptosis within the tumor eventually leads to a higher incidence of pre-treatment resistance rather than what would be expected based on the size of the tumor only [53]. Moreover, our study suggests that oxidative stress-associated cell death, observed in many other chronic conditions [54] may be directly linked to tumorigenesis through associated increase in cell survival.

In conclusion, oxidized extracellular DNA released by dying tumor cells may stimulate survival of tumor cells. Importantly, in cells exposed to oxidized DNA, a suppression of cell death is accompanied by an increase in the markers of genome instability. Survival of cells with an unstable genome may substantially augment progression of malignancy. The model that describes the role of oxidized DNA released from apoptotic cells in tumor biology is depicted in Figure 12. 

## Materials and Methods

### Cell culture

ER/PR-positive MCF-7 breast cancer cells were purchased at ATCC, Manassas, USA (Cat: HTB-22). Human embryonic lung fibroblasts were retrieved from the biospecimen collection maintained by the Research Centre for Medical Genetics, Russian Academy of Medical Sciences collection and grown as described in [7]. Ethical approval for the use of primary human cells was obtained from the Committee for Medical and Health Research Ethics of Research Centre for Medical Genetics, Russian Academy of Medical Sciences (2012, approval number 5). 

MCF-7 cells were cultured in DMEM medium supplemented with 10% (v/v) fetal calf serum, 2 mM L-glutamine, 100 units/mL penicillin, and 100 μg/mL of streptomycin. Cells were grown in a humidified atmosphere with 5% CO2 in air at 37°C. Before treatment with DNA probes, cells were grown for 24 h or 72 h in slide flasks.

### Flow cytomery

Before flow cytometry, cells were washed in Versene solution, than treated with 0.25 % trypsin under control of light microscopic observation. Cells were transferred to the Eppendorf tubes, washed with culture media, then centrifuged and resuspended in PBS. Staining of the cells with various antibodies was performed as described below. Briefly, to fix the cells, the paraformaldehyde (Sigma) was added at a final concentration of 2 % at 37°C for 10 min. Cells were washed three times with 0.5% BSA-PBS and permeabilized with 0.1% Triton X-100 (Sigma) in PBS for 15 min or with 70% ethanol at 4°C. Cells (~ 50 x 10^3^) were washed three times with 0.5% BSA-PBS and stained with 1 - 2 μg/mL FITC-γH2AX (Ser139) antibody (Temecula California), FITC-Ki-67 antibody, PCNA, 8-oxodG, EEA1, AIM2, TLR9, NRF2, NF-κB (p65) , S529 NF-κB (p65) and STAT3 antibodies (Abcam) for 3 h at 4°C, then again washed thrice with 0.5% BSA-PBS and stained with 1 μg/mL secondary FITC-conjugated or PE-conjugated antibodies (Abcam) for 1 h at 4°C. To quantify intracellular DNA, cells were treated with propidium iodide and RNAase A. To quantify the background fluorescence, we stained a portion of the cells with secondary FITC(PE)-conjugated antibodies only. Cells were analyzed at CyFlow Space (Partec, Germany).

#### Annexin V binding assays

Following treatment with gDNA or gDNA^OX^, cells were detached by trypsinization, counted and pelleted (1000 r.p.m. for 5 min). Cell pellets were washed once with PBS and once in Annexin V binding buffer (10 mM HEPES, pH 7.4, 140 mM NaCl, 2.5 mM CaCl2). Cells were treated with Annexin V-FITC at room temperature for 15 min in the dark. Cells were analyzed for fluorescence on CyFlow Space.

### Fluorescent microscopy

Cell images were obtained using the AxioScope A1 microscope (Carl Zeiss).

#### Immunocytochemistry

MCF-7 cells were fixed in 3% formaldehyde (4°C) for 20 min, washed with PBS and then permeabilized with 0.1% Triton X-100 in PBS for 15 min at room temperature, followed by blocking with 0.5% BSA in PBS for 1 h and incubated overnight at 4°C with the FITC-γH2AX (Ser139), 8-oxodG, NRF2, STAT3, NF-κB (p65), AIM2 antibody. After washing with 0.01% Triton X-100 in PBS MCF-7 cells were incubated for 2 h at room temperature with the FITC/PE goat anti-mouse IgG, washed with PBS and then stained with DAPI. 

#### Intracelullar localization of labeled DNA fragments

Labeled fractions of gDNA-Red, gDNA^Red-OX^ and pBR322^Green^ (50 ng/ml) were added to cultivation media for 30 min. Cells were washed three times with PBS, fixed in 3% paraformaldehyde (4°C) for 20 min, washed with PBS and stained with 2 μg/mL DAPI. To analyze distribution of 8-oxodG, MCF-7 cells were permeabilized with 0.1% Triton X-100 in PBS for 15 min at room temperature, then treated with respective antibodies.

#### Analysis of genomic instability

Before treatment with DNA probes, cells were grown for 24 h or 72 h in slide flasks. The DNA fractions were added to cultivation media for 24 hours. Cells were fixed in 3% formaldehyde (4°C) for 20 min, washed with PBS and stained with 2 μg/mL DAPI. Approximately 2,000 cells were investigated for the presence of micronuclei, nuclear buds and nuclear bridges as described by Fenech (2009).

#### Nuclear fragmentation

Was examined by Hoechst 33342 (Sigma) staining (10 μg/mL) for 10 min at 37°C. 1,000 cells were investigated for the presence of the damaged nuclei. 

#### ROS detection assays

Cells were grown in slide flasks and treated in two different protocols [1]. MCF-7 cell cultures were pretreated with 5μM of H_2_DCFH-DA (Molecular Probes/Invitrogen, CA, USA) for 5 min, then ecDNA samples were added for further 30 min; (2) ecDNA samples were added to MCF-7 cultures, cell were grown for 1 hour, then cells were treated with 5μM of H_2_DCFH-DA for 30 min. In both cases, cells were washed three times with PBS and immediately photographed. 

#### Mitochondria

In cells were stained with 30 nM TMRM (tetramethylrhodamine methyl ester) (Molecular Probes) for 20 min at 37°C. 

### Extraction of the DNA fragments from the cells or the cell-free media

To extract extracellular DNA, cells were removed from the media by centrifugation at 460 x g, followed by mixing of 3 mL of the media with 0.3 mL of the solution containing 1% sodium lauryl sarcosylate, 0.02 M EDTA, and 75 μg/mL RNAse A (Sigma, USA), incubation for 45 min, then the 24-h treatment with proteinase K (200 μg/mL, Promega, USA) at 37°C. Intact gDNA was extracted from primary human embryonic fibroblasts (HEFs) [7]. To extract genomic DNA, cells separated, and the DNA was extracted form lysed cells. After two cycles of the purification with saturated phenolic solution, DNA fragments were precipitated by adding two volumes of ethanol in the presence of 2M ammonium acetate. The precipitate was then washed with 75% ethanol twice, then dried and dissolved in water. The concentration of DNA was determined by measuring fluorescence intensity after DNA staining with the RiboGreen (Molecular Probes/Invitrogen, CA, USA). Mean size of untreated gDNA fragments was 30 kb. To match gDNA and gDNA^OX^ samples in its mean size, gDNA was hydrolyzed by DNAse I until size distribution of its fragments became from 0.2 to 15 kb. 

### Generation of the DNA samples

#### gDNA^ox^.

gDNA solution (100 ng/mL) was combined with H_2_O_2_ (300 mM) under UV light (312 nm) for 30 min, 25 °C [15]. Modified DNA was precipitated with 2 volumes of ethanol in the presence of 2 M ammonium acetate, then washed twice with 75% ethanol, dried and dissolved in water. Resulting DNA concentrations were assessed by the analysis of the UV spectra. The size distribution of its gDNA^OX^ fragments was from 0.2 to 15 kb. 

#### gDNA^red^ and pBR322^green^

Labeling of extracted genomic and plasmid DNA was performed by nick translation using CGH Nick Translation Kit (Abbott Molecular) under manufacturer’s protocol with slight modification. Solutions of genomic human and plasmid DNA (3 µg/µL) were labeled with SpectrumRed and SpectrumGreen, respectively. In the reaction mix, 50% of the dTTP was substituted with the labeled dUTP. About 20% of the fluorescent-labeled nucleotide was incorporated into the DNA, while unincorporated nucleotides were removed by ethanol precipitation. The fragment size was in 300–3000 bp range as determined by electrophoresis in 1% agarose.

#### gDNA^red-OX^.

gDNA^red^ (100 ng/ml) and gDNA^ox^ (100 ng/ml) were heated to 75°С in 70% formamide-PBS and slowly cooled to 42°C using the StepOne Plus (Applied Biosystems), then kept at 37°С for a few hours.

### Quantification of mRNA levels

Total mRNA was isolated from cells using RNeasy Mini kit (Qiagen, Germany). After the treatment with DNAse I, RNA samples were reverse transcribed by Reverse Transcriptase kit (Sileks, Russia). The expression profiles were obtained using quantitative reverse transcriptase polymerase chain reaction (qRT-PCR) with SYBRgreen PCR MasterMix (Applied Biosystems). Three housekeeping genes, ACTB, GADPH and TBP, were evaluated as possible reference genes in MCF-7 exposed to oxidized DNA. An expression of TBP was found the most stable and the employed as reference standard in further experiments. The mRNA levels were analyzed in several independent experiments using the StepOne Plus (Applied Biosystems); the technical error (%CV) was approximately 2%. All PCR products were run in the polyacrylamide gel (PAGE) to confirm their size. The following primers were used (Sintol, Russia):

*AIM2* (F: CAGAAATGATGTCGCAAAGCAA, R: TCAGTACCATAACTGGCAAACAG)

*BCL2* (F:GCCTTCTTTGAGTTCGGTGG, R: ATCTCCCGGTTGACGCTCT)

*BCL2A1* (Bfl-1/A1) (F:TACAGGCTGGCTCAGGACTAT, R: CGCAACATTTTGTAGCACTCTG)

*BCL2L1* (BCL-X) (F:CGACGAGTTTGAACTGCGGTA, R: GGGATGTCAGGTCACTGAATG)

*BIRC3* (c-IAP1) (F:AAGCTACCTCTCAGCCTACTTT, R: CCACTGTTTTCTGTACCCGGA)

*BMP2* (F:ACTACCAGAAACGAGTGGGAA, R: CATCTGTTCTCGGAAAACCTGAA)

*BMP4* (F:AAAGTCGCCGAGATTCAGGG, R: GACGGCACTCTTGCTAGGC)

*BRCA1* (F:TGTGAGGCACCTGTGGTGA, R: CAGCTCCTGGCACTGGTAGAG)

*CDKN2A* (p16INK4) (F:ATGGAGCCTTCGGCTGACT, R: TAACTATTCGGTGCGTTGGG)

*CDKN1A* (p21CIP1/WAF1) (F:GGAAGACCATGTGGACCTGT, R: ATGCCCAGCACTCTTAGGAA)

*FOS* (F:GGGGCAAGGTGGAACAGTTAT, R: CCGCTTGGAGTGTATCAGTCA)

*GATA-4* (F:GCCCAAGAACCTGAATAAATCTAAG, R: AGACATCGCACTGACTGAGAACGTC)

*ICAM1* (F:CGTGCCGCACTGAACTGGAC, R: CCTCACACTTCACTGTCACCT)

*IL10* (F:AAGGCGCATGTGAACTCCC, R: ACGGCCTTGCTCTTGTTTTC)

*IL6* (F:AAATTCGGTACATCCTCGACGGCA, R: AGTGCCTCTTTGCTGCTTTCACAC)

*IL8* (F:ACTGAGAGTGATTGAGAGTGGAC, R: AACCCTCTGCACCCAGTTTTC)

*JUN* (F:TCCAAGTGCCGAAAAAGGAAG, R: CGAGTTCTGAGCTTTCAAGGT)

*KEAP1* (F:GTGGTGTCCATTGAGGGTATCC, R : GCTCAGCGAAGTTGGCGAT)

*MAP4K4* (F:GAGCCACAGGTACAGTGGTC, R: AAGCCTTTTGGGTAGGGTCAG)

*MAPK8* (JNK1) (F:AGAAGCTAAGCCGACCATTTC, R: TCTAGGGATTTCTGTGGTGTGA)

*MYD88* (F: GGCTGCTCTCAACATGCGA, R: TGTCCGCACGTTCAAGAACA);

*NANOG* (F:GCTGAGATGCCTCACACGGAG, R: TCTGTTTCTTGACTGGGACCTTGTC); 

*NFKB1*(F:CAGATGGCCCATACCTTCAAAT, R: CGGAAACGAAATCCTCTCTGTT);

*NRF2* (*NFE2L2*) (F:TCCAGTCAGAAACCAGTGGAT, R: GAATGTCTGCGCCAAAAGCTG);

*OCT4* (F:TGGAGAAGGAGAAGCTGGAGCAAAA, R: GGCAGATGGTCGTTTGGCTGAATA);

*PECAM1* (F:CCAAGGTGGGATCGTGAGG, R: TCGGAAGGATAAAACGCGGTC);

*RHOA* (F:TGGAAAGACATGCTTGCTCAT, R: GCCTCAGGCGATCATAATCTTC);

*RIG1* (F:GAGATTTTCCGCCTTGGCTAT, R: CCGTTTCACCTCTGCACTGTT); 

*SELE* (F:CAGCAAAGGTACACACACCTG, R: CAGACCCACACATTGTTGACTT);

*SELP* (F:CAGACCACTCAACCAGCAG, R: GGCCGTCAGTCGAGTTGTC);

*STAT3* (F:GGGTGGAGAAGGACATCAGCGGTAA, R: GCCGACAATACTTTCCGAATGC); 

*STAT6* (F:GTTCCGCCACTTGCCAATG, R: TGGATCTCCCCTACTCGGTG);

*STING* (F: CCAGAGCACACTCTCCGGTA, R: CGCATTTGGGAGGGAGTAGTA);

*TIRAP* (F:ATGGTGGCTTTCGTCAAGTCA, R: TCAGATACTGTAGCTGAATCCCG);

*TLR9* (F: CCCACCTGTCACTCAAGTACA, R: GTGGCTGAAGGTATCGGGATG);

*TP53* (F:TTTGGGTCTTTGAACCCTTG, R: CCACAACAAAACACCAGTGC);

*TNFa* (F: CAGCCTCTTCTCCTTCCTGAT, R: GCCAGAGGGCTGATTAGAGA);

*VCAM1* (F:GGGAAGCCGATCACAGTCAAG, R: AAATTCGGTACATCCTCGACGGCA);

*VEGFA* (F:AGGCCAGCACATAGGAGAGA, R: TTTCTTGCGCTTTCGTTTTT);

*TBP* (reference gene) (F: GCCCGAAACGCCGAATAT, R: CCGTGGTTCGTGGCTCTCT).

### Blocking ROS

Some experiments were supplemented with controls exposed to both oxidized DNA and antioxidant N-acetyl-cysteine (NAC) at 0.15 mM. In these cases, NAC was added to the media 30 minutes before exposure to DNA.

### Statistics

All reported results were reproduced at least three times as independent biological replicates. In FACS, the mean values of signal intensities were analyzed. The Figures show the average data and the standard deviation (SD). The significance of the observed differences was analyzed using non-parametric Mann-Whitney U-tests. P-values < 0.05 were considered statistically significant и marked at Figures with (*). Data were analyzed with StatPlus2007 Professional software (http://www.analystsoft.com).
